# Ubiquitination of the DNA-damage checkpoint kinase CHK1 by TRAF4 is required for CHK1 activation

**DOI:** 10.1186/s13045-020-00869-3

**Published:** 2020-05-01

**Authors:** Xinfang Yu, Wei Li, Haidan Liu, Qipan Deng, Xu Wang, Hui Hu, Zijun Y. Xu-Monette, Wei Xiong, Zhongxin Lu, Ken H. Young, Wei Wang, Yong Li

**Affiliations:** 1 Department of Medicine, Baylor College of Medicine, Houston, TX USA; 2 Department of Cancer Biology, Lerner Research Institute, Cleveland Clinic, Cleveland, OH USA; 3 Department of Radiology, The Third Xiangya Hospital of Central South University, Changsha, Hunan China; 4 Clinical Center for Gene Diagnosis and Therapy, The Second Xiangya Hospital of Central South University, Changsha, Hunan China; 5 Department of Medical Laboratory, Central Hospital of Wuhan, Wuhan, Hubei China; 6 Department of Hematopathology, The University of Texas MD Anderson Cancer Center, Houston, TX USA; 7 Cancer Research Institute and School of Basic Medical Science, Xiangya School of Medicine, Central South University, Changsha, Hunan China

**Keywords:** Colorectal cancer, Checkpoint kinase 1, Tumor necrosis factor receptor-associated factor 4, Ubiquitination, Chemoresistance

## Abstract

**Background:**

Aberrant activation of DNA damage response (DDR) is a major cause of chemoresistance in colorectal cancer (CRC). CHK1 is upregulated in CRC and contributes to therapeutic resistance. We investigated the upstream signaling pathways governing CHK1 activation in CRC.

**Methods:**

We identified CHK1-binding proteins by mass spectrometry analysis. We analyzed the biologic consequences of knockout or overexpression of TRAF4 using immunoblotting, immunoprecipitation, and immunofluorescence. CHK1 and TRAF4 ubiquitination was studied in vitro and in vivo. We tested the functions of TRAF4 in CHK1 phosphorylation and CRC chemoresistance by measuring cell viability and proliferation, anchorage-dependent and -independent cell growth, and mouse xenograft tumorigenesis. We analyzed human CRC specimens by immunohistochemistry.

**Results:**

TRAF4 catalyzed the ubiquitination of CHK1 in multiple CRC cell lines. Following DNA damage, ubiquitination of CHK1 at K132 by TRAF4 is required for CHK1 phosphorylation and activation mediated by ATR. Notably, TRAF4 was highly expressed in chemotherapy-resistant CRC specimens and positively correlated with phosphorylated CHK1. Furthermore, depletion of TRAF4 impaired CHK1 activity and sensitized CRC cells to fluorouracil and other chemotherapeutic agents in vitro and in vivo.

**Conclusions:**

These data reveal two novel steps required for CHK1 activation in which TRAF4 serves as a critical intermediary and suggest that inhibition of the ATR–TRAF4–CHK1 signaling may overcome CRC chemoresistance.

## Introduction

Colorectal cancer (CRC) is the third most common cancer and the second leading cause of cancer-related death worldwide [1]. Currently, fluorouracil (5-Fu)-based chemotherapy is one of the most frequently used therapeutic strategies for advanced CRC patients, and its mechanism of action is direct or indirect induction of DNA damage response (DDR) pathways [2]. Although the majority of patients with advanced CRC are initially responsive to the 5-Fu-based combination chemotherapy, tumors frequently recur due to drug resistance. Immune checkpoint inhibitors have recently shown promise in a limited subset of patients as a treatment for CRC [3]. Therefore, further elucidating the mechanisms of DDR and chemoresistance in CRC and improving the efficacy of chemotherapy have important clinical significance.

DNA-damage checkpoints are signaling cascades that either trigger cell cycle arrest, which allows time for DNA repair or induce apoptotic cell death [4]. Transduction of the DDR signal, such as that induced by 5-Fu, is often carried out by a set of conserved checkpoint protein kinases, including ataxia telangiectasia mutated (ATM), ATM- and Rad3-related (ATR) proteins, and the downstream checkpoint kinases CHK2 and CHK1. Phosphorylation of CHK1 at S317 and S345 by the ATR kinase is required for its activation in response to DNA damage [4]. CHK1 is upregulated in multiple human cancers, including CRC, and its overexpression and/or hyperactivation is linked to chemo- and radiotherapy resistance [5–10]. CHK1-targeted therapy selectively eliminates replication-stressed, p53-deficient, and hyperdiploid colorectal cancer stem cells [11, 12]. Accumulating evidence has shown that CHK1 ubiquitination is critical for its stability, subcellular localization, and phosphorylation. Furthermore, K48-linked ubiquitination downregulates CHK1 expression, whereas K63-linked ubiquitination upregulates CHK1 activity [13–15]. However, how the DDR triggers the ubiquitination-dependent activation of CHK1 to promote CRC cell survival remains elusive.

Tumor necrosis factor receptor-associated factor 4 (TRAF4, also known as RING finger protein 83 or RNF83) is emerging as a critical regulator for cancer cell proliferation, survival, and metastasis [16–18]. TRAF4 promotes the invasion of CRC cells by regulating Wnt/β-catenin signaling [19, 20]. Recent reports suggest the N-terminal RING finger domain of TRAF4 has E3 ligase activity that ubiquitinates AKT, SMURF2, and TrkA to promote tumor growth and metastasis [16–18]. Due to the critical role of TRAF4 in tumorigenesis and its druggable enzymatic activity, identification of novel TRAF4 substrates will be helpful for developing new cancer treatment strategies.

Here, we identified TRAF4 as an E3 ligase that ubiquitinates CHK1. During DDR, TRAF4 ubiquitinates CHK1, which is a prerequisite for CHK1 chromatin association and subsequent phosphorylation and activation by ATR in CRC cells. The absence of TRAF4 sensitizes CRC cells to chemotherapeutic agents in a CHK1 ubiquitination- and activation-dependent manner, suggesting that CHK1 ubiquitination by TRAF4 is a potential therapeutic target to overcome CRC chemoresistance.

## Materials And methods

### Cell culture and reagents

HT29, SW620, HCT-8, FHC, HeLa, U2OS, A549, H1975, and 293T cells were obtained from the American Type Culture Collection (ATCC, Manassas, VA, USA) and cultured at 37 °C in a humidified incubator with 5% CO_2_ according to ATCC protocols. All cell lines were routinely checked for mycoplasma and tested cytogenetically to ensure their authentication. Chemical reagents, including Tris, NaCl, SDS, and dimethyl sulfoxide (DMSO), were purchased from Sigma-Aldrich (St. Louis, MO, USA). Oxaliplatin (cat. #S1224), 5-Fu (cat. #S1209), irinotecan (cat. #S2217), cisplatin (cat. #S1166), pemetrexed (cat. #S1135), gemcitabine (cat. #S1714), VE-821 (cat. #S8007), AZ20 (cat. #S7050), and prexasertib were obtained from Selleck Chemicals (Houston, TX, USA). Lipofectamine™ 2000 (cat. #11668019, Thermo Fisher Scientific) transfection reagent was used for transient transfection experiments following the manufacturer’s instruction. Ctrl siRNA (cat. #sc-93314), UbcH6 siRNA (cat. #sc-61744), BTG3 siRNA (cat. #sc-43644), and ATR siRNA (cat. #sc-29763) were purchased from Santa Cruz Biotechnology (Dallas, TX, USA). Three independent experiments were completed for each cell line, treatment, and time point, as indicated.

### Immunoblotting (IB) and immunoprecipitation (IP)

Whole-cell lysates were extracted with RIPA buffer (cat. #89900, Thermo Fisher Scientific) supplemented with phosphatase inhibitors (cat. #78428, Thermo Fisher Scientific). The lysates were sonicated and centrifuged at 12000×*g* for 15 min at 4 °C. The BCA Assay Reagent (cat. #23228, Thermo Fisher Scientific) was used to determine protein concentration. For co-immunoprecipitation (co-IP) assays, cells were lysed with IP Lysis Buffer (cat. #87787, Thermo Fisher Scientific). IB and co-IP were performed as previously described [16]. All antibodies for IB analysis were diluted in phosphate-buffered saline (PBS) buffer with 5% non-fat milk. Antibodies against Bax (cat. #5023; IB, 1:1000), Bik (cat. #4592; IB, 1:1000), Bim (cat. #2933; IB, 1:1000), Bid (cat. #2002; IB, 1:1000), Bak (cat. #12105; IB, 1:1000), survivin (cat. #2808; IB, 1:1000), Bcl-2 (cat. #4223; IB, 1:1000), Bcl-xL (cat. #2764; IB, 1:1000), Mcl-1 (cat. #5453; IB, 1:1000), γ-H2AX (cat. #9718; IB, 1:4000), α-tubulin (cat. #2144; IB, 1:10000), ubiquitin (cat. #3936; IB, 1:1000), cleaved-caspase 3 (cat. #9664; IB, 1:2000), cleaved-PARP (cat. #5625; IB, 1:2000), p-(Ser/Thr) ATM/ATR substrate (cat. #2851; IB, 1:1000), p-ATR (S428) (cat. # 2853; IB, 1:1000), p-ATR (Thr1989) (cat. #30632; IB, 1:1000), ATR (cat. # 13934; IB, 1:1000), p-CHK1 (S317) (cat. #12302; IB, 1:1000), p-CHK1 (S345) (cat. #2348; IB, 1:1000), CHK1 (cat. #2360; IB, 1:1000; IP, 1:200), p-CDC25C (Ser216) (cat. #4901; IB, 1:1000), CDC25C (cat. #4688; IB, 1:1000), GST tag (cat. #2624; IB, 1:5000; IP, 1:200), K63-linkage-specific polyubiquitin (cat. #12930; IB, 1:1000), rabbit IgG HRP (cat. #7074; IB, 1:10000), and mouse IgG HRP (cat. #7076; IB, 1:10,000) were obtained from Cell Signaling Technology, Inc. (Danvers, MA, USA). Antibodies against β-actin (cat. #A5316; IB, 1:10000), TRAF4 (cat. #MABC985; IB, 1:4000; IP, 1:200), Flag tag (cat. #F3165; IB, 1:10000; IP, 1:400), and Flag–HRP (cat. #A8592; IB, 1:20000) were from Sigma-Aldrich (St. Louis, MO, USA). Antibodies against HA tag (cat. #ab18181; IB, 1:5000; IP, 1:200) and His tag (cat. #ab18184; IB, 1:5000) were purchased from Abcam (Cambridge, UK). GFP-tag (cat. #TA150032; IB, 1:4000; IP, 1:400) antibody was obtained from OriGene (Rockville, MD, USA). Rabbit anti-TRAF4 (cat. #A302-840A; IB, 1:1000; IP, 1:200) and anti-CHK1 (cat. #A300-298A; IB, 1:1000; IP, 1:200) antibodies were purchased from Bethyl Laboratories (Montgomery, TX, USA). Antibody conjugates were visualized by chemiluminescence (cat. #34076, Thermo Fisher Scientific).

### Plasmid construction

*Flag–TRAF4* (cat. #RC200345), *GFP–TRAF4* (cat. #RC200345L4), *Flag–Chk1* (cat. #RC205094), and *GFP–Chk1* (cat. #RC225807L4) were obtained from OriGene. *pCDNA3–HA–Akt1* (cat. #73408) was obtained from Addgene (Watertown, MA, USA). *His–Ub* was a gift from Jianneng Li at Lerner Research Institute, Cleveland Clinic. *Flag–Chk1* (DM-N), *Flag–Chk1* (DM-C), *Flag–TRAF4* (DM-RING), *Flag–TRAF4* (DM-Inter), and *Flag–TRAF4* (DM-TRAF), *Flag–GFP–TRAF4* (C18A), *Flag–GFP–TRAF4* (T192A), *Flag–GFP-–TRAF4* (T192D), *His–Ub* (K6, K11, K27,K29, K33, K48, and K63), *His–Ub* (K48R), *His–Ub* (K63R), and *Flag–Chk1* (S317/345A, K38R, K54R, K145R, K132R, K233R, K244R, K404R, K444R, K451R, and K456/458R) mutants were developed using the Q5 Site-Directed Mutagenesis Kit (cat. #E0554S, NEB) following the manufacturer’s protocol. All mutant constructs were generated using mutagenesis PCR were verified by Sanger DNA sequencing.

### CRISPR-Cas9-mediated knockout assays

To generate CRISPR-Cas9-based *TRAF4* and *Chk1* knockout constructs, we cloned the annealed single-guide RNAs (sgRNAs) into the Bsm BI-digested lentiCRISPR V2 vector (cat. #52961, Addgene). The sgRNAs were from the Human CRISPR Knockout Pooled Library (GeCKO v2) [21] and are listed as follows: sg*TRAF4*#1 forward, 5′-AGCCACAAAACTCGCACTTG-3′; reverse, 5′-CAAGTGCGAGTTTTGTGGCT-3′; sg*TRAF4*#2 forward, 5′-CTCTGCCCATTCAAAGACTC-3′; reverse, 5′-GAGTCTTTGAATGGGCAGAG-3′; sg*Chk1*#1 forward, 5′-AGTCATGGCAGTGCCCTTTG-3′; reverse, 5′-CAAAGGGCACTGCCATGACT-3′; sg*Chk1*#2 forward, 5′-GAGATTCTTCCATCAACTCA-3′; reverse, 5′-TGAGTTGATGGAAGAATCTC-3′. The control or stable knockout cells were generated by transient transfection of sgRNA-inserted CRISPR-Cas9 plasmids and selected with 2 μg/ml puromycin for 2–3 weeks.

### Cell proliferation assays

Cells were seeded at a density of 3 × 10^3^ cells/well in 96-well plates. After overnight incubation, fresh medium containing different doses of chemotherapeutic agents was added, and the plates were maintained for 0, 24, 48, or 72 h. The 3-(4,5-dimethylthiazol-2-yl)-5-(3-carboxymethoxyphenyl)-2-(4-sulfophenyl)-2H-tetrazolium (MTS) reagent (cat. #G3581, Promega) was used for cell viability assays. Cell proliferation was measured using the 5-ethynyl-2′-deoxyuridine (EdU) assay. Briefly, cells were seeded at a density of 1 × 10^4^ cells in chamber slides (cat. #PEZGS0416, Millipore) and cultured overnight. Cells were then treated with 10 μM 5-Fu for 24 h. Next, 10 μM EdU (cat. #C10339, Thermo Fisher Scientific) was added to the culture medium, which was incubated for an additional 12 or 24 h. Cells were fixed and stained following the manufacturer’s instructions.

### Anchorage-independent cell growth assay

The soft-agar colony formation assay was performed as previously described [16]. Briefly, CRC cells were treated with chemotherapeutic agents or treated for 24 h, suspended (1 × 10^4^ cells per well) in 1 mL of 0.3% agar with Eagle’s basal medium containing 10% FBS, and overlaid into six-well plates containing a 0.6% agar base. The cultures were maintained at 37 °C in a 5% CO_2_ incubator, and the colonies were counted at 2 weeks.

### Plate colony formation assay

Cells were treated with chemotherapeutic agents or their vehicle control for 24 h and seeded into a 6-cm plate (300 cells/well). Cells were maintained for 2 weeks at 37 °C in a 5% CO_2_ incubator. Colonies were fixed with 4% paraformaldehyde, stained with 0.5% crystal violet, and counted under a microscope. Three independent experiments were performed as indicated.

### In vitro kinase assay

For the CHK1 IP/kinase assay, stable HT29 cells were treated with 5-Fu or UV light and lysed in IP buffer containing protease and phosphatase inhibitors. CHK1 was immunoprecipitated from cell lysates with an anti-CHK1 antibody (cat. #A300-298A, Bethyl Laboratories) at 4 °C for 4 h and washed twice in IP buffer and twice in 1× kinase buffer (cat. #9802, Cell Signaling Technology, Inc.). The recombinant GST–CDC25C protein (1 μg) was mixed with the immunoprecipitated CHK1 in a 20 μL reaction and incubated with 100 μM ATP in 1× kinase buffer at 30 °C for 30 min. Protein phosphorylation was examined by IB.

### Mass spectrometry

293T cells stably expressing Flag–CHK1 were lysed in an IP buffer containing protease and phosphatase inhibitors. IP was performed overnight at 4 °C with the whole-cell lysates using the Flag-tag antibody (cat. #F3165, Sigma-Aldrich) or normal mouse IgG (cat. #5415, Cell Signaling Technology). The IP proteins were resolved by SDS-PAGE, followed by Coomassie blue staining. The desired proteins were then digested and subjected to LC-MS at the Cleveland Clinic Proteomics Core. The peptides were analyzed using all collected collision-induced dissociation (CID) spectra and the search programs Sequest and Mascot. Protein and peptide identifications were validated using the program Scaffold.

### In vivo ubiquitination assay

Cells were lysed with a lysis buffer (6 M guanidine-HCl, 0.1 M Na_2_HPO_4_/NaH_2_PO_4_, 0.01 M Tris/HCl, pH 8.0, 5 mM imidazole, and 10 mM β-mercaptoethanol) supplemented with protease inhibitors and 10 mM N-ethylmaleimide (NEM, cat. #S3692, Selleck Chemicals). After sonication and centrifugation, the supernatant was incubated at room temperature for 4 h with 40 μL Ni-NTA-agarose beads (cat. #30210, Qiagen Inc.). The beads were centrifuged and washed sequentially with five buffers: (A) 6 M guanidine-HCl, 0.1 M Na_2_HPO_4_/NaH_2_PO_4_, 0.01 M Tris/HCl, pH 8.0, and 5 mM imidazole plus 10 mM β-mercaptoethanol; (B) 8 M urea, 0.1 M Na_2_HPO_4_/NaH_2_PO_4_, 0.01 M Tris/HCl, pH 8.0, 10 mM imidazole, and 10 mM β-mercaptoethanol plus 0.1% Triton X-100; (C) 8 M urea, 0.1 M Na_2_HPO_4_/NaH_2_PO_4_, 0.01 M Tris/HCl, pH 6.3, 10 mM β-mercaptoethanol, and 20 mM imidazole plus 0.2% Triton X-100; (D) 8 M urea, 0.1 M Na_2_HPO_4_/NaH_2_PO_4_, 0.01 M Tris/HCl, pH 6.3, 10 mM β-mercaptoethanol, and 10 mM imidazole plus 0.1% Triton X-100; and (E) 8 M urea, 0.1 M Na_2_HPO_4_/NaH_2_PO_4_, 0.01 M Tris/HCl, pH 6.3, 10 mM β-mercaptoethanol, 10 mM imidazole plus 0.05% Triton X-100. After the last wash, the beads were boiled with 2× SDS sample-loading buffer containing 200 mM imidazole, and the supernatant was separated by SDS-PAGE, followed by IB.

### IP-mediated endogenous ubiquitination assay

Cells were lysed with a modified RIPA buffer (20 mM NAP, pH 7.4, 150 mM NaCl, 1% Triton X-100, 0.5% sodium deoxycholate, and 1% SDS) supplemented with protease inhibitors (cat. #78430, Thermo Fisher Scientific) and 10 mM NEM. After sonication, the lysates were boiled at 95 °C for 15 min, diluted with RIPA buffer containing 0.1% SDS, then centrifuged at 1.6 × 10^4^×*g* for 10 min at 4 °C. The supernatant was incubated overnight at 4 °C with the primary antibodies and 40 μL protein A-Sepharose beads. After washing with RIPA buffer, the beads were boiled with 2× SDS sample-loading buffer to elute the bound protein. The eluted protein was then separated by SDS-PAGE, followed by IB. The Chromatin Extraction Kit (ab117152, Abcam) was used for chromatin and non-chromatin fractions extraction following the standard instruction.

### In vitro ubiquitination assay

The in vitro ubiquitination assay was performed as previously described [17]. Briefly, Flag–TRAF4, Flag–TRAF4 (DM-RING), and Flag–TRAF4 (C18A) were expressed in 293T cells, immunoprecipitated with anti-Flag antibody, and eluted with Flag peptide. Flag–TRAF4, Flag–TRAF4 (DM-RING), or Flag–TRAF4 (C18A) protein along with GST–CHK1 protein (cat. #14-346, Millipore) were incubated with 40 nM Ube1 (E1), 0.7 μM UbcH6 (E2), and 10 μM ubiquitin for 3 h at 37 °C in reaction buffer (50 mM Tris-HCl, pH 7.5, 5 mM MgCl_2_, 1 mM DTT, and 2 mM ATP). After incubation, the protein mixtures were diluted with RIPA buffer and immunoprecipitated overnight with the anti-CHK1 antibody at 4 °C. CHK1 ubiquitination was then analyzed by IB.

### Xenograft mouse model

All procedures for the xenograft mouse models were conducted under protocols approved by our Institutional Animal Care and Use Committee (IACUC). Xenograft tumors were established by subcutaneous injection of stable HT29, SW620, and H1975 cells (1 × 10^6^) into the right flank of 6-week-old NSG mice (The Jackson Laboratory, Bar Harbor, ME, USA). Tumors were measured with calipers every 2days, and mice received one of the following treatments by intraperitoneal injection when tumors reached a volume of 100 mm^3^: (1) vehicle, every 4 days; (2) 5-Fu, 50 mg/kg every 4 days; (3) oxaliplatin, 5 mg/kg every 4 days; (4) irinotecan, 10 mg/kg every 4 days; (5) cisplatin, 5 mg/kg every 4 days; or subcutaneous injection of (6) prexasertib, 16 mg/kg twice every 7 days; (7) the combination of prexasertib, 16 mg/kg twice every 7 days, and 5-Fu, 50 mg/kg every 4 days. Tumor volume was calculated with the following formula: tumor volume (mm^3^) = (length × width × width/2), where length is the longest diameter and width is the shortest diameter. Mice were euthanized at the endpoint, and xenografted tumors were dissected and analyzed.

### Clinical tissue sample collection

CRC patients were diagnosed and classified by the Department of Pathology at The Third Xiangya Hospital and Xiangya Hospital according to the World Health Organization guidelines. All surgical specimens were collected in accordance with an Institutional Review Board-approved protocol. All subjects provided written informed consent for entry into this study. Individuals included 92 cases of primary adenocarcinomas with matched adjacent tissue, 22 cases of CRC liver metastasis, 12 cases of CRC lung metastasis, and 23 cases of relapse after 5-Fu + oxaliplatin + leucovorin combination chemotherapy.

### Immunohistochemical (IHC) staining

Tumor tissues obtained from CRC patients or xenografts were subjected to hematoxylin and eosin (H&E) staining or IHC staining with specific antibodies. Briefly, the tissue sections were baked at 60 °C for 2 h, deparaffinized, and rehydrated. Slides were submerged into boiling sodium citrate buffer (10 mM, pH 6.0) for 10 min and incubated in 3% H_2_O_2_ in methanol for 10 min. Slides were blocked with 50% goat serum albumin for 1 h at room temperature and incubated with primary antibodies overnight in a cold room in a humidified chamber. Slides were washed with PBS, followed by incubation with the secondary antibody for 1 h at room temperature. Hematoxylin was used for counterstaining, which was evaluated independently by two pathologists. The percentage of positive cells was scored as follows: 0, no positive cells; 1, ≤ 10% positive cells; 2, 10–50% positive cells; and 3, > 50% positive cells. Staining intensity was scored as follows: 0, no staining; 1, faint staining; 2, moderate staining; and 3, dark staining. Comprehensive score = staining percentage × intensity. For TRAF4/p-CHK1 expression, a comprehensive score ≤ 1.5 is considered low expression and > 1.5 high expression. The association between TRAF4/p-CHK1 levels and patient clinical features, including age, gender, initial clinical stage, tumor stage, and lymph node status, is summarized in Supplementary Table 4 and Supplementary Table 5. The following antibodies were used for IHC staining: Ki67 (cat. #ab16667, Abcam, 1:250), TRAF4 (cat. #MABC985, Sigma-Aldrich, 1:300), p-CHK1 (cat. #ab47318, 1:200), and CHK1 (cat. #ab47574, 1:100). The Click-iT™ TUNEL Colorimetric IHC Detection Kit (cat. #c10623, Thermo Fisher Scientific) was used for apoptosis detection in tissue sections according to the manufacturers’ standard procedures.

### Immunofluorescence (IF)

Cells in a chamber slide were treated with 10 μM 5Fu for 24 h. The cells were fixed with 4% paraformaldehyde and permeabilized in 0.5% Triton X-100 for 20 min, followed by blocking in 5% BSA for 1 h and overnight incubation with γ-H2AX or p-CHK1 primary antibodies at 4 °C in a humidified chamber. Alexa Fluor 488 dye-labeled anti-rabbit IgG was used as the secondary antibody. Nuclei were counterstained with DAPI (cat. #P36935, Thermo Fisher Scientific). For measuring chemotherapeutic agent-induced γ-H2AX or p-CHK1, cells were seeded onto a chamber slide and treated with the drugs overnight. The chemotherapeutic agent-free medium was then added to the chamber slide, and the cells were fixed at various time points (0, 24, or 48 h) as indicated. The following antibodies were used for IF: γ-H2AX (cat. #ab11174, 1:100), p-CHK1 (cat. #ab47318, 1:200), α-tubulin (cat. #ab7291, 1:100), γ-tubulin (cat. #ab11317, 1:100), goat anti-rabbit IgG Alexa Fluor 488 (cat. #ab150077, 1:500), goat anti-mouse IgG Alexa Fluor 488 (cat. #ab150117, 1:500), and donkey anti-rabbit IgG Alexa Fluor Dye 647 (cat. #ab150075, 1:500).

### Statistical analysis

Statistical analyses were performed using SPSS (version 16.0 for Windows, SPSS Inc., Chicago, IL, USA) and GraphPad Prism (GraphPad 7.0, San Diego, CA, USA). All quantitative data are expressed as the mean ± standard error of the mean (s.e.m) from 3 independent experiments. Differences between means were evaluated with Student’s *t* test or analysis of variance (ANOVA). The clinicopathologic significance of clinical samples was evaluated with a *χ*^2^ test or a Fisher exact test for categorical data. The Mann–Whitney *U* test was used when the data did not fit a normal distribution. Kaplan–Meier analysis and the log-rank test (Mantel–Cox) were used for survival analysis. Pearson rank correlation was used for correlation tests. The Wilcoxon matched-pair signed-rank test was used for evaluating expression differences between tumors and adjacent non-cancerous tissues. A probability value of *p* ≤ 0.05 was used as the criterion for statistical significance.

## Results

### CHK1 activation is regulated by the TRAF4 E3 ligase in response to DNA damage

**Fig. 1:**
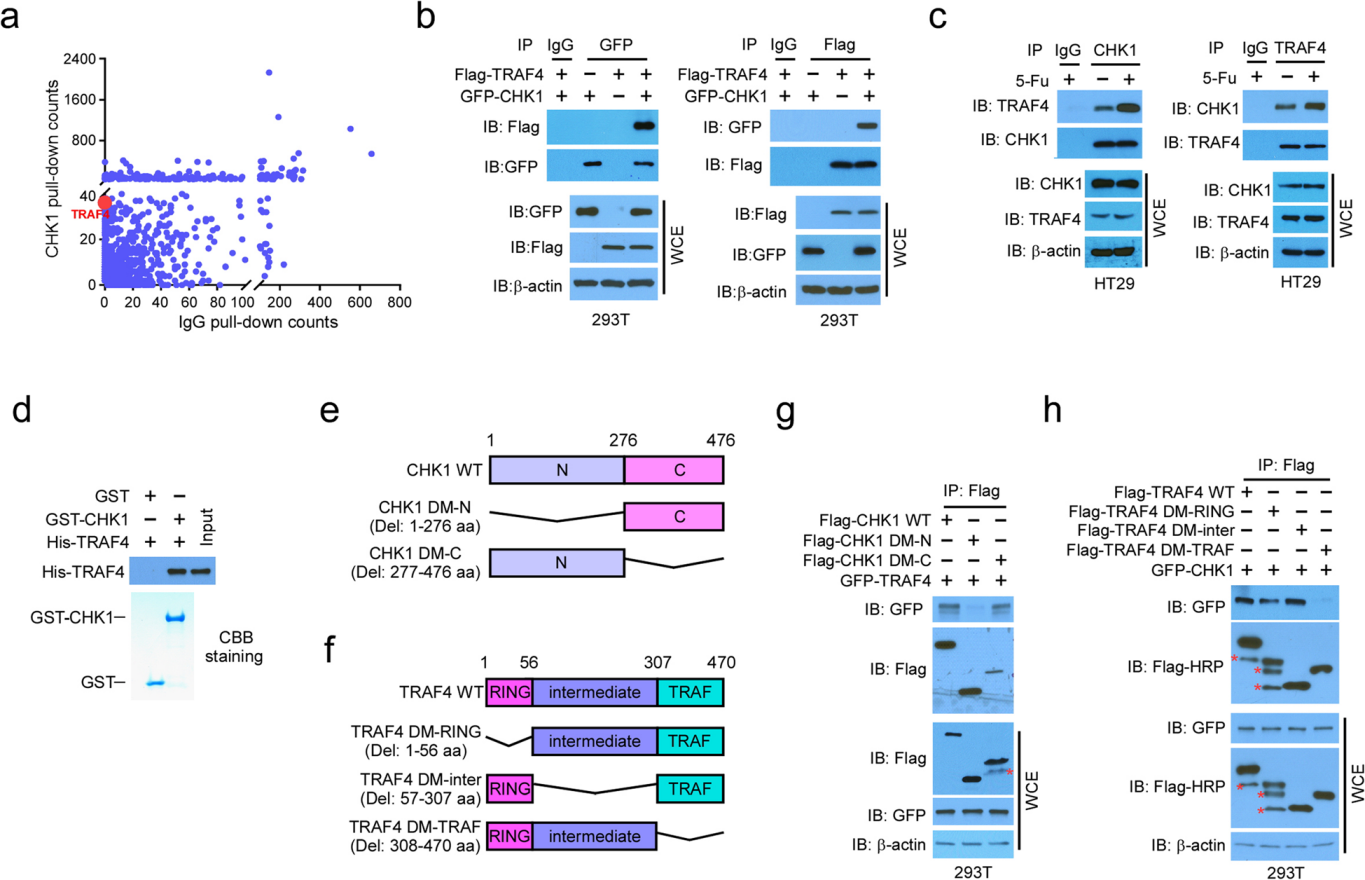
CHK1 interacts with TRAF4. **a** Scatterplot showing the proteins pulled down by CHK1 and Control IgG antibodies. **b** Co-immunoprecipitation (Co-IP) analysis of the TRAF4 and CHK1 interaction in 293T cells transfected with the indicated constructs. WCE, whole-cell extract. **c** Co-IP analysis of the endogenous TRAF4-CHK1 interaction in HT29 cells. **d** Cell-free binding between TRAF4 and CHK1. **e, f** Schematic of CHK1 WT and deletion mutants (**e**) and TRAF4 WT and deletion mutants (**f**) used in this study. N, N-terminal; C, C-terminal; RING, RING domain; Intermediate, zinc finger and coiled-coil domain; TRAF, TRAF-C domain. **g** Co-IP analysis of TRAF4 and CHK1 truncation mutant interactions. 293T cells were transfected with the indicated constructs and subjected to Co-IP and immunoblotting (IB) analysis. **h** Co-IP analysis of CHK1 and TRAF4 truncation mutant interactions. 293T cells were transfected with the indicated constructs and subjected to Co-IP and IB analysis. *Non-specific signal

To better understand the regulation of CHK1 activation, we performed a mass spectrometry analysis to identify novel CHK1-binding proteins in 293T cells stably expressing Flag-CHK1. In addition to known CHK1-associated proteins, including nucleic acid binding-related proteins and hydrolases, protein ubiquitin E3 ligases, including TRAF4, were identified as major interacting partners of CHK1 compared to the normal IgG negative control (Fig. 1a). The interaction between CHK1 and TRAF4 was confirmed by co-immunoprecipitation (Co-IP) of ectopically expressed proteins in 293T cells (Fig. 1b), and endogenous binding was confirmed via reciprocal IP in both HT29 (Fig. 1c) and SW620 (Fig. S1a) CRC cells. We further confirmed the direct binding of CHK1 and TRAF4 in vitro with purified protein samples (Fig. 1d). Deletion of the N-terminal domain of CHK1 significantly impaired the CHK1-TRAF4 interaction (Fig. 1e, g), while the C-terminal TRAF domain was essential for TRAF4 to bind to CHK1 (Fig. 1f, h). The endogenous interaction between TRAF4 and CHK1 was enhanced in HT29 cells that were treated with various chemotherapeutic agents (Fig. S1b).

**Fig. 2:**
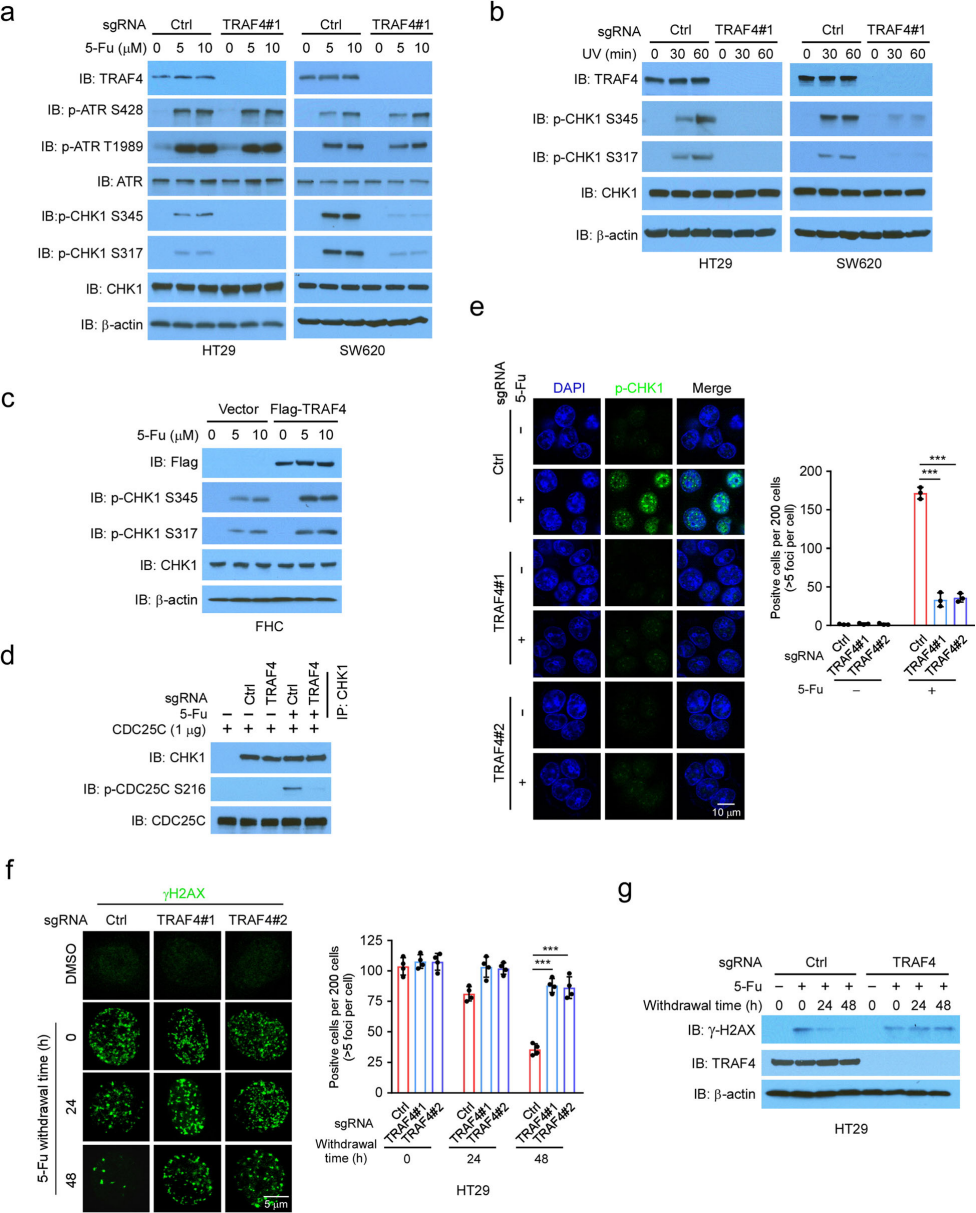
CHK1 activation is regulated by TRAF4. **a** TRAF4 knockout reduces CHK1 phosphorylation in 5-Fu-treated CRC cells. HT29 and SW620 cells expressing sgCtrl or sgTRAF4 were treated with 5-Fu for 24 h. WCEs were subjected to IB analysis. **b** TRAF4 knockout inhibits CHK1 phosphorylation in UV-treated CRC cells. HT29 and SW620 cells expressing sgCtrl or sgTRAF4 were treated with UV. WCEs were collected at various time points and subjected to IB analysis. **c** TRAF4 overexpression enhances CHK1 phosphorylation in 5-Fu-treated FHC cells. IB analysis of immortalized colon epithelial cell FHC transfected with vector control or Flag-TRAF4 constructs and treated with 5-Fu. **d** TRAF4 knockout reduces CHK1 kinase activity ex vivo. TRAF4-WT or TRAF4-knockout HT29 cells were treated with 5-Fu for 24 h, and the WCEs were prepared and immunoprecipitated with the CHK1 antibody. The immunoprecipitated CHK1 was incubated with recombinant CDC25C and subjected to an in vitro kinase assay. **e** The IF analysis of CHK1 phosphorylation in 5-Fu-treated HT29 cells. ***p < 0.001. Scale bar, 10 μm. **f**, **g** TRAF4 knockout compromises DNA repair in 5-Fu-treated HT29 cells. TRAF4-WT or TRAF4-knockout HT29 cells were treated with 5-Fu for 24 h, then maintained in 5-Fu-free medium and subjected to IF (**f**) or IB (**g**) analysis at various time points with γ-H2AX antibody. ***p < 0.001. Scale bar, 5 μm

Next, we determined whether TRAF4 was required for DNA damage-induced CHK1 activation in CRC cells. Strikingly, 5-Fu-induced phosphorylation of CHK1 at S345 and S317 was drastically decreased in TRAF4-knockout HT29 and SW620 cells with 2 independent sgRNAs when compared to control cells (Fig. 2a, Fig. S1c). However, TRAF4 knockout had no effect on 5-Fu-induced ATR activation (Fig. 2a). A similar reduction in CHK1 phosphorylation was observed in TRAF4-knockout cells treated with oxaliplatin (Fig. S1d) or irinotecan (Fig. S1e), which are two other first-line chemotherapeutic agents for CRC. Consistently, overexpression of TRAF4 enhanced CHK1 phosphorylation induced by 5-Fu in non-cancerous colon epithelial FHC cells, which expressed low levels of endogenous TRAF4 (Fig. 2c). Additionally, an in vitro kinase assay showed that the immunoprecipitated CHK1 from 5-Fu-treated TRAF4-knockout CRC cells exhibited decreased kinase activity (Fig. 2d), which was measured by the phosphorylation of CDC25C, a CHK1 substrate [22]. Moreover, 5-Fu treatment induced phosphorylation of CHK1 at the S345 (p-CHK1) foci in the nucleus of wild-type (WT) HT29 cells but not in TRAF4-knockout HT29 cells (Fig. 2e). Immunofluorescence (IF) staining revealed that exposure to 5-Fu for 24 h significantly enhanced γ-H2AX foci formation in both TRAF4-WT HT29 and TRAF4-knockout HT29 cells. However, TRAF4 deficiency decreased the efficacy of DNA damage repair as γ-H2AX foci formation after 5-Fu withdrawal was significantly reduced in TRAF4-WT HT29 cells but not in TRAF4-knockout HT29 and SW620 cells (Fig. 2f, Fig. S1f). Immunoblotting also revealed that γ-H2AX protein levels were higher in TRAF4-knockout cells when compared to TRAF4-proficient HT29 and SW620 cells (Fig. 2g, Fig. S1g). Similar results of the γ-H2AX foci formation were obtained in HT29 cells treated with oxaliplatin and irinotecan (Fig. S1h and S1i).

Because activation of CHK1 is required to prevent premature mitotic entry [23], we hypothesized that TRAF4 regulated mitosis by promoting CHK1 phosphorylation in CRC. In support of this hypothesis, over 95% of mitotic HT29 cells in the control group developed a bipolar mitotic spindle assembly during mitosis after 5-Fu treatment. In contrast, a loss of TRAF4 led to ~ 45% of 5-Fu-exposed mitotic cells with monopolar or multipolar spindles, chromosome misalignment during metaphase, or chromosome lagging during anaphase (Fig. S2a and 2b). Moreover, TRAF4 deficiency resulted in upregulation of the polyploidy cell population in 5-Fu-treated HT29 cells, and these polyploidy cells had decreased 5-ethynyl-2′-deoxyuridine (EdU) incorporation (Fig. S2c). Our results are consistent with the phenotypes for premature mitotic progression and mitotic catastrophe in CHK1-deficient or inactivated cells [23, 24]. These results support the framework that TRAF4 is required for CHK1 phosphorylation and activation in response to DNA damage.

### CHK1 K63-linked ubiquitination is mediated by TRAF4

**Fig. 3:**
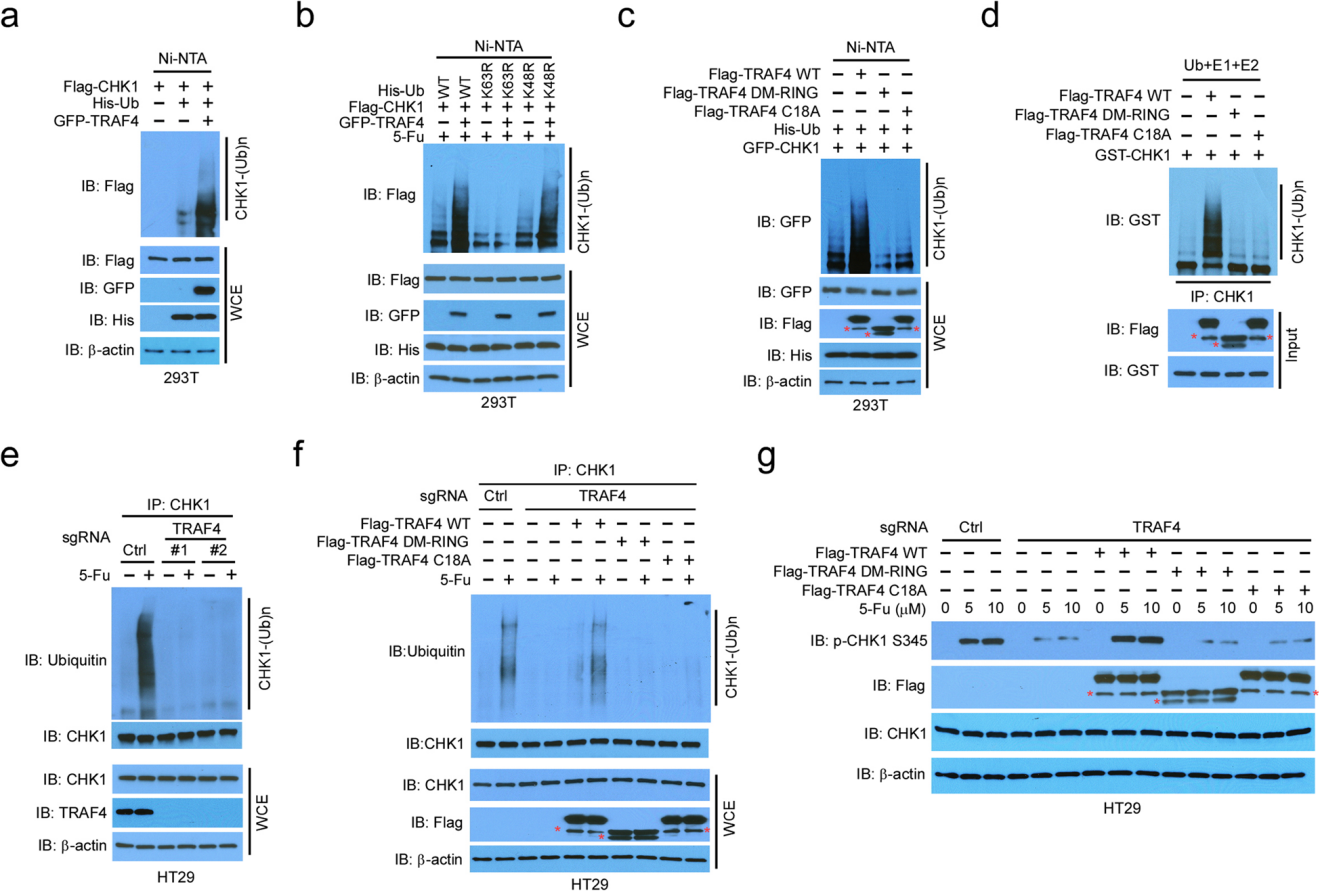
TRAF4 promotes K63-linked ubiquitination of CHK1 is required for CHK1 activation. **a** In vivo CHK1 ubiquitination by TRAF4 in 293T cells transfected with various constructs. **b** CHK1 ubiquitination by TRAF4 is K63-linked. An in vivo ubiquitination assay was performed in 293T cells transfected with various constructs and treated with 5-Fu. **c** In vivo CHK1 ubiquitination by TRAF4 mutants. TRAF4 WT, TRAF4 (DM-RING) mutant, or TRAF4 (C18A) mutant were co-transfected into 293T cells with various constructs, and WCEs were subjected to an in vivo ubiquitination assay. **d** In vitro CHK1 ubiquitination by TRAF4. **e** Endogenous CHK1 ubiquitination by TRAF4 in HT29 cells. TRAF4-WT or TRAF4-knockout HT29 cells were treated with 5-Fu for 24 h, and WCEs were immunoprecipitated and subjected to ubiquitination analysis. **f** CHK1 ubiquitination in TRAF4-null cells is rescued by exogenous WT TRAF4. TRAF4-knockout HT29 cells were transfected with various constructs and treated with 5-Fu, and WCEs were immunoprecipitated for IB. **g** CHK1 phosphorylation in TRAF4-null cells is rescued by exogenous WT TRAF4. TRAF4-knockout HT29 cells were transfected with various constructs and treated with different doses of 5-Fu, and WCEs were immunoblotted. *Non-specific signal

Next, we investigated the molecular mechanisms by which TRAF4 promoted CHK1 activation. As depletion of TRAF4 had a negligible effect on ATR phosphorylation, but significantly impaired CHK1 activation (Fig. 2a), TRAF4 appeared to be a downstream target of ATR. We hypothesized that TRAF4 functioned as an E3 ligase to mediate CHK1 activation through ubiquitination. TRAF4 promoted CHK1 ubiquitination in 293T cells (Fig. 3b). Notably, the proteasome inhibitor MG132 did not enhance this process substantially (Fig. S3a), suggesting that the ubiquitination did not cause protein degradation. Consistent with this finding, substitution of WT ubiquitin with a mutant K63R markedly suppressed TRAF4-induced CHK1 ubiquitination (Fig. 3b). Moreover, treatment with 5-Fu facilitated the ubiquitination of endogenous CHK1 in SW620 cells (Fig. S3b). Although WT TRAF4 overexpression promoted CHK1 ubiquitination, the activities of the E3 ligase-defective TRAF4 mutants, DM-RING and C18A, were compromised in 293T cells (Fig. 3c). Interestingly, the binding of the DM-RING and C18A mutants to CHK1 was unaffected (Fig. S3c). An in vitro ubiquitination assay suggested that CHK1 was a direct target of TRAF4, but not the DM-RING and C18A TRAF4 mutants (Fig. 3d). Moreover, silencing of UbcH6, the ubiquitin-conjugating enzyme (E2), which is critical for TRAF4 E3 ligase activity [17], impaired TRAF4-mediated CHK1 ubiquitination (Fig. S3d). In addition, 5-Fu-stimulated endogenous CHK1 ubiquitination was diminished in TRAF4-deficient CRC HT29 and SW620 cells (Fig. 3e, Fig. S3e). To corroborate the role of TRAF4 in CHK1 ubiquitination, we introduced WT TRAF4 and its E3 ligase activity-deficient mutants into TRAF4-knockout HT29 cells. As shown in Fig. 3f 5-Fu-induced CHK1 ubiquitination was restored with WT TRAF4, but not with the DM-RING mutant or the catalytically inactive C18A mutant. Moreover, WT TRAF4 rescued 5-Fu- or UV-induced CHK1 phosphorylation (Fig. 3g, Fig. S3f) and 5-Fu-induced p-CHK1 foci formation (Fig. S3g). These data underscore the importance of TRAF4-mediated CHK1 ubiquitination to CHK1 phosphorylation and activation in CRC cells. Collectively, these results support that TRAF4 is a E3 ligase that controls CHK1 ubiquitination in CRC cells.

### CHK1 activation depends on K132 ubiquitination

**Fig. 4:**
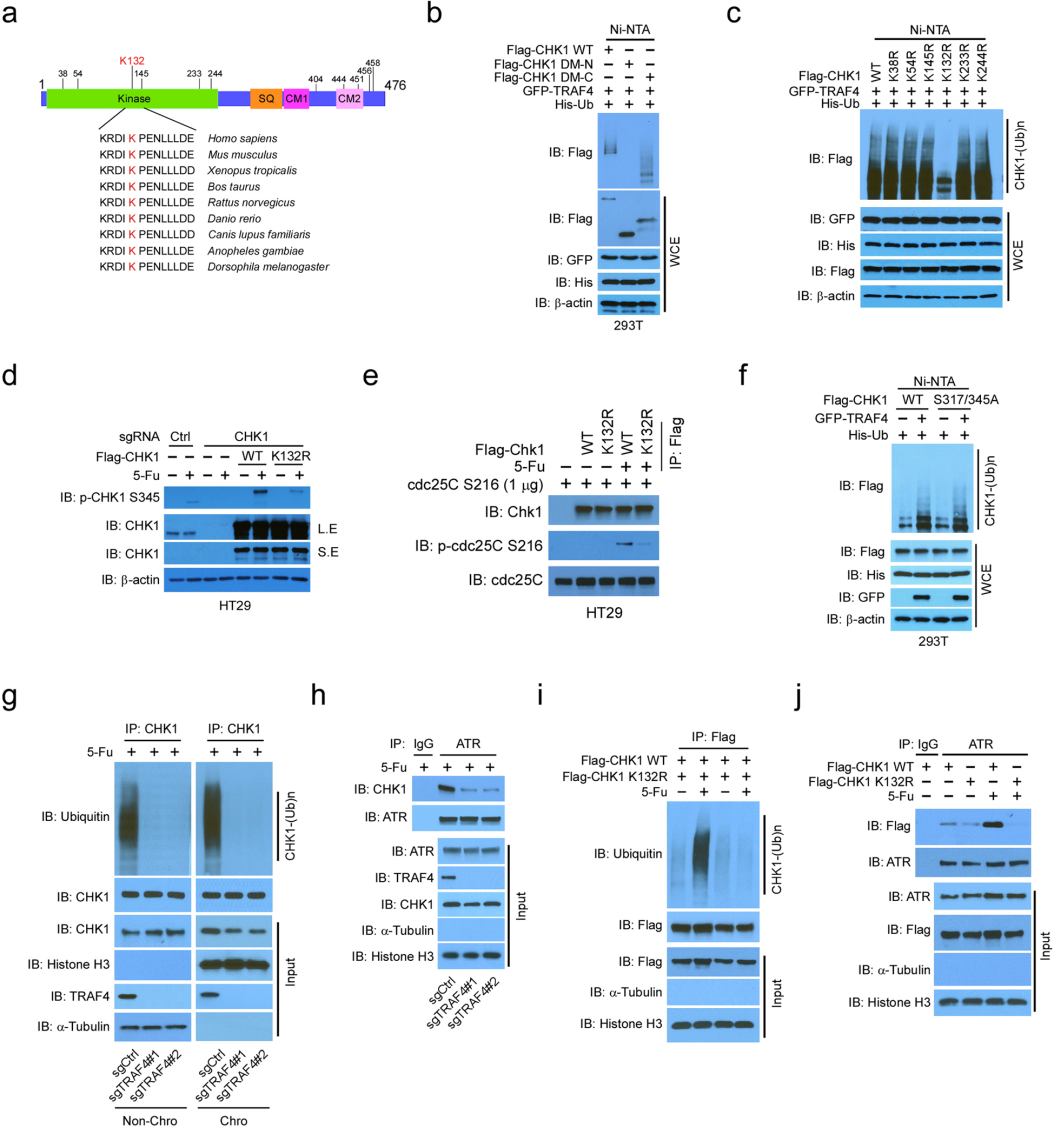
CHK1 K132 was ubiquitinated by TRAF4. **a** Alignment of conserved CHK1 lysine residues among different species. Kinase, kinase domain; SQ, Ser/Gln cluster; CM, conserved motif. **b** Ubiquitination of CHK1 mutants by TRAF4 was determined by an in vivo ubiquitination assay in 293T cells transfected with various constructs. **c** Lysine (K) 132 is the ubiquitination site in CHK1. 293T cells were transfected with various constructs and subjected to in vivo ubiquitination analysis. **d** CHK1 phosphorylation in CHK1-null HT29 cells is rescued by exogenous WT CHK1. CHK1 WT or CHK1 knockout HT29 cells transfected with CHK1 WT or CHK1 K132R mutant were treated with 5-Fu and subjected to IB analysis. **e** In vitro kinase assay of WT and mutant CHK1. HT29 cells were transfected with various constructs and treated with 5-Fu. WCEs were immunoprecipitated with Flag-tag antibody. The IP proteins were incubated with CDC25C and subjected to an in vitro kinase assay. **f** In vivo ubiquitination of WT CHK1 and the S317/345 mutant by TRAF4 in 293T cells with 5-Fu treatment. **g** Chromatin-associated CHK1 ubiquitination in HT29 cells with 5-Fu treatment. TRAF4-WT or TRAF4-knockout HT29 cells were treated with 5-Fu for 24 h, and chromatin and non-chromatin fractions were isolated and subjected to immunoprecipitated and ubiquitination analysis. Chro, chromatin faction. Non-Chro, cytoplasmic proteins and soluble nuclear proteins. **h** Co-IP analysis of ATR and CHK1 interaction in chromatin fraction. TRAF4-WT or TRAF4-knockout HT29 cells were treated with 5-Fu for 24 h, and chromatin fraction was isolated and subjected to Co-IP analysis. **i** Chromatin-associated CHK1 WT and K132R mutant ubiquitination in HT29 cells with 5-Fu treatment. HT29 cells were transfected with Flag-CHK1 or Flag-CHK1 K132R and treated with 5-Fu for 24 h, and chromatin fraction was isolated and subjected to immunoprecipitated and ubiquitination analysis. **j** Co-IP analysis of ATR and CHK1 interaction in chromatin fraction. HT29 cells were transfected with Flag-CHK1 or Flag-CHK1 K132R and treated with 5-Fu for 24 h, and chromatin fraction was isolated and subjected to Co-IP analysis

To identify CHK1 ubiquitination sites, we analyzed the evolutionarily conserved lysine residues among CHK1 proteins from different species. The 11 most highly conserved sites were primarily localized in its N-terminal kinase domain and the C-terminal conserved motif 2 (CM2) (Fig. 4a). In vivo ubiquitination assays demonstrated that TRAF4 promoted the ubiquitination of both WT CHK1 and mutant CHK1 with a C-terminal deletion, but not mutant CHK1 with an N-terminal deletion in 293T cells (Fig. 4b). These data suggested that the ubiquitination sites are localized in the N-terminal kinase domain of CHK1. Using single-site mutants of CHK1, we discovered that only the mutation of K132 to arginine (R) markedly attenuated CHK1 ubiquitination in 293T cells (Fig. 4c). We therefore focused on CHK1 K132 ubiquitination and investigated its role in CHK1 activation. The K132R mutation caused a robust decrease in 5-Fu-induced CHK1 ubiquitination in HT29 cells (Fig. S4a). Moreover, 5-Fu-induced phosphorylation of S317 phosphorylation was reduced in HT29 cells exogenously expressing the K132R CHK1 mutant compared to that with WT CHK1 (Fig. S4b). Reconstitution of WT CHK1, but not the K132R mutant, restored 5-Fu-induced CHK1 S345 phosphorylation in CHK1-knockout HT29 and SW620 cells (Fig. 4d, Fig. S4c). In addition, in vitro kinase assays showed that K132R substantially impaired CHK1-mediated CDC25C phosphorylation in HT29 treated with 5-Fu (Fig. 4e) or in 293T cells treated with UV (Fig. S4d). A previous study suggested that the adaptor protein B-cell translocation gene 3 (BTG3) is required for UV-induced CHK1 ubiquitination in HCT116 cells [13]. We found that BTG3 or TRAF4 silencing decreased CHK1 ubiquitination and that TRAF4 knockdown more effectively inhibited CHK1 ubiquitination when compared to BTG3 knockdown in HT29 cells (Fig. S4e). To determine whether phosphorylation of CHK1 at S345 and S317 was required for ubiquitination, a CHK1 (S317/345A) double mutant was constructed for an in vivo ubiquitination assay in 293T cells. 5-Fu failed to induce phosphorylation of the S317/345A mutant (Fig. S4f), yet TRAF4-mediated ubiquitination of WT CHK1 was similar to that of the S317/345A mutant (Fig. 4f). These results suggest that TRAF4 promotes CHK1 ubiquitination independent of phosphorylation at S317/345, whereas phosphorylation at S317/345 is dependent on CHK1 ubiquitination.

To determine how ubiquitination regulates CHK1 phosphorylation, we isolated the chromatin fraction of HT29 cells treated with 5-Fu. Given that chromatin binding is required for ATR kinase-mediated CHK1 phosphorylation, we examined whether ubiquitination enhanced CHK1 chromatin association. Depletion of TRAF4 robustly attenuated 5-Fu-induced CHK1 ubiquitination on chromatin (Fig. 4g) and in non-chromatin fractions (cytoplasmic proteins and soluble nuclear proteins). Moreover, the total protein levels of CHK1 in the chromatin fraction were decreased in TRAF4-knockout HT29 cells (Fig. 4g), indicating that TRAF4-deficient impaired 5-Fu induced CHK1 ubiquitination and chromatin association. In addition, the Co-IP result indicated that the interaction between ATR and CHK1 in chromatin fraction was decreased in TRAF4-knockout HT29 cells (Fig. 4h). Consistently, mutation of the major ubiquitination site, K132R, reduced chromatin-associated CHK1 ubiquitination and CHK1 expression in response to 5-Fu treatment (Fig. 4i). Loss of ubiquitination on K132 impaired 5-Fu-induced ATR and CHK1 interaction (Fig. 4j). These results indicate that the ubiquitination of CHK1 on K132 is required for DNA damage-induced CHK1 chromatin association and subsequent ATR interaction and activation.

### TRAF4 deficiency impairs CHK1 activity and confers sensitivity to chemotherapy in CRC cells

**Fig. 5:**
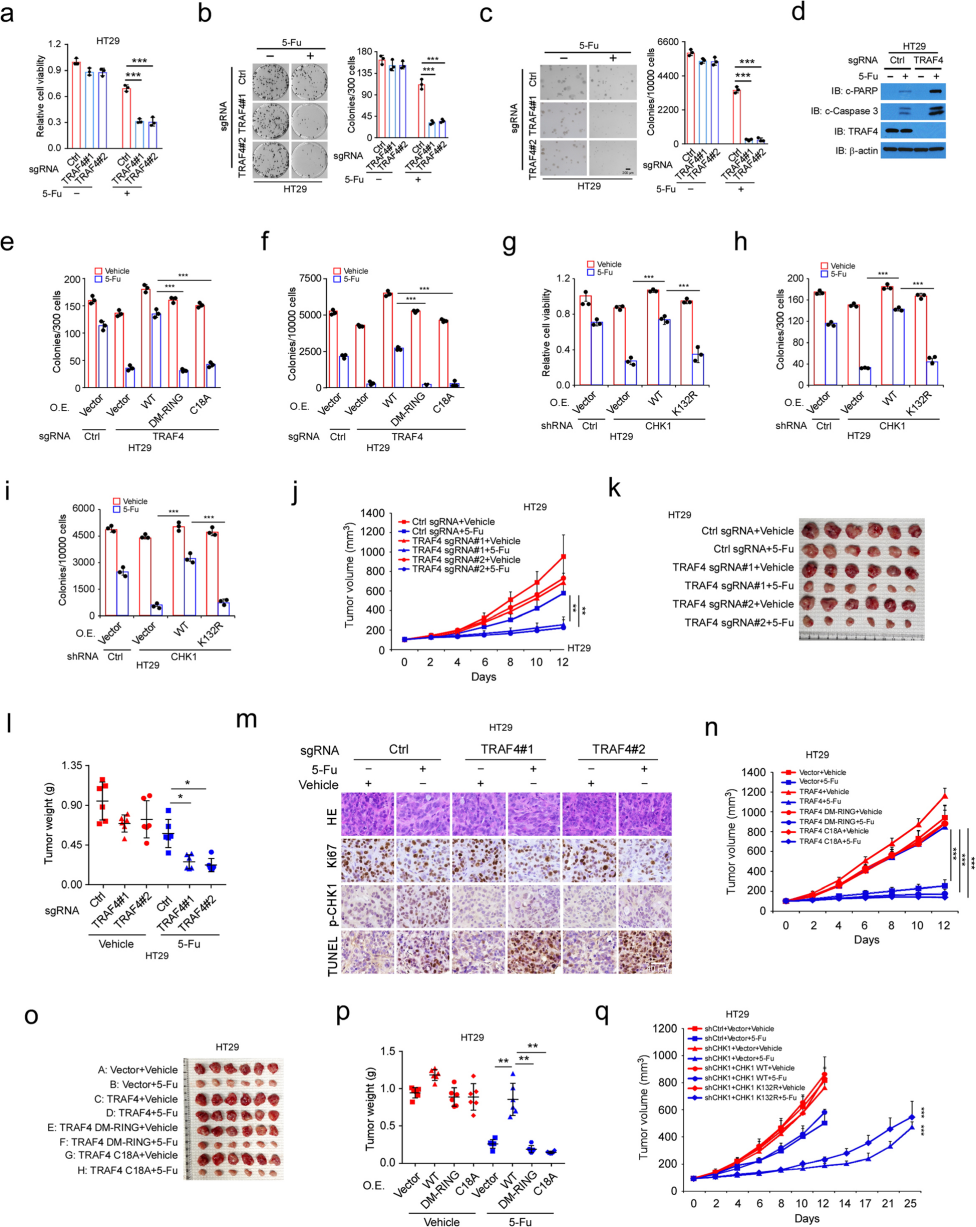
TRAF4 deficiency impairs CHK1 activity and confers sensitivity to chemotherapy in CRC cells. **a** TRAF4 knockout reduces cell viability in HT29 cells with 5-Fu treatment. HT29 cells expressing sgCtrl or sgTRAF4 were treated with 5-Fu for 72 h and analyzed with the MTS assay. ***p < 0.001. **b, c** Anchorage-dependent (**b**) and -independent (**c**) growth of TRAF4-WT or TRAF4-knockout HT29 cells with 5-Fu treatment. ***p < 0.001. **d** TRAF4 knockout enhances 5-Fu-induced apoptosis. TRAF4-WT or TRAF4-knockout HT29 cells were treated with 5-Fu for 72 h, and WCEs were immunoblotted. **e, f** Anchorage-dependent (**e**) and -independent (**f**) growth of TRAF4-null HT29 cells rescued by exogenous WT TRAF4. ***p < 0.001. **g-i **Cell viability (**g**) and anchorage-dependent (**h**) and -independent (**i**) growth of CHK1 knockdown HT29 cells rescued by exogenous WT CHK1. ***p < 0.001. **j**–**m** TRAF4 knockout enhances the efficacy of 5-Fu in vivo. Tumor size was monitored (**j**). Tumors were dissected (**k**) and weighed (**l**). Ki67 and p-CHK1 were examined via IHC staining, and apoptosis was analyzed with the TUNEL assay (**m**). *p < 0.05, **p < 0.01. **n**–**p** TRAF4 reintroduction into TRAF4-null HT29 cells rescues tumorigenesis under 5-Fu treatment. TRAF4 WT or E3 ligase activity-deficient mutants were reintroduced into TRAF4-null HT29 cells and injected into NSG mice to establish the xenograft mouse model. Tumor size was monitored (**n**). Tumors were dissected (**o**) and weighed (**p**). **p < 0.01, ***p < 0.001. **q** CHK1 reintroduction into CHK1-knockdown HT29 cells rescues tumorigenesis under 5-Fu treatment. WT CHK1 or CHK1 K132R mutant was reintroduced into CHK1-knockdown HT29 cells and injected into NSG mice to establish the xenograft mouse model. Mice were treated with 5-Fu, and tumor size was monitored. ***p < 0.001

We next investigated whether TRAF4 affects the sensitivity of human CRC cells to chemotherapy. TRAF4 knockout attenuated cell viability in HT29 and SW620 cells in the presence of 5-Fu (Fig. 5a, Fig. S5a). The anchorage-dependent and -independent colony formation potential of both TRAF4-knockout HT29 and SW620 cells was impaired in the presence of 5-Fu (Fig. 5b, c; Fig. S5b and S5c). Similar inhibitory effects were observed in HT29 cells treated with other chemotherapeutic agents, including oxaliplatin and irinotecan (Fig. S5d and S5e). TRAF4 knockout in HT29 cells enhanced chemotherapy-induced apoptosis, which was indicated by the upregulation of cleaved-caspase 3 and -PARP (Fig. 5d). Flow cytometry data showed that the apoptotic cell population was increased in 5-Fu-treated TRAF4-knockout HT29 cells (Fig. S5f); consistently, the comet assay revealed that knockout of TRAF4 enhanced 5-Fu-induced DNA damage (Fig. S5g). These results suggest that TRAF4 deletion promotes the anti-tumor effects of chemotherapeutic agents. Restoration of WT TRAF4, but not the E3 ligase-inactive mutants DM-RING and C18A, promoted the colony formation and anchorage-independent cell growth of TRAF4-knockout HT29 cells receiving 5-Fu treatment (Fig. 5e, f). This in vitro evidence implies that the TRAF4 E3 ligase activity is required for TRAF4-mediated desensitization of CRC cells to chemotherapy. To determine whether CHK1 K132 ubiquitination was required for chemoresistance in CRC cells with high TRAF4 expression, we introduced WT CHK1 or the CHK1 K132R mutant into HT29 cells with stable CHK1 knockdown. Reconstitution of WT CHK1, but not the ubiquitination-deficient K132R mutant, attenuated 5-Fu-induced suppression of cell viability (Fig. 5g) and anchorage-dependent and -independent colony formation (Fig. 5h, i).

Xenograft tumors derived from TRAF4-knockout HT29 cells were treated with 5-Fu and exhibited a significant decrease in tumor growth, tumor mass, and tumor cell proliferation when compared to tumors derived from TRAF4-knockout cells that did not receive the 5-Fu treatment or to tumors retaining WT TRAF4 and treated with 5-Fu (Fig. 5j–m). Moreover, 5-Fu promoted CHK1 phosphorylation in WT-TRAF4 xenograft tumors but attenuated in TRAF4-knockout xenograft tumors (Fig. 5m). In addition, 5-Fu induced increased apoptosis in TRAF4-knockout xenograft tumors when compared to WT-TRAF4 xenograft tumors (Fig. 5m). Similar results were observed in tumors derived from SW620 cells (Fig. S5h and S5i). Other chemotherapeutic agents, such as oxaliplatin and irinotecan, also inhibited TRAF4-knockout tumor development from HT29 cells (Fig. S5j–m). In the TRAF4-knockout HT29 tumors, reintroduction of WT TRAF4 impaired the anti-tumor effectiveness of the chemotherapeutic agents, but reintroduction of the DM-RING and C18A TRAF4 mutants, which both lack the E3 ligase activity, did not (Fig. 5n–p, Fig. S5n). We next determined the chemotherapeutic function of CHK1 K132 ubiquitination in vivo. Xenograft tumors derived from CHK1-knockdown HT29 cells that were treated with 5-Fu exhibited reduced tumor growth (Fig. 5q). Reconstitution of WT CHK1, but not the ubiquitination-deficient mutant CHK1 K132R, attenuated the anti-tumor efficacy of 5-Fu (Fig. 5q). These results suggest that TRAF4 deletion or deficient TRAF4 E3 ligase activity restricts CHK1 activation and inhibits xenograft tumorigenesis during CRC chemotherapy, and that CHK1 K132 ubiquitination is required for maintaining chemoresistance in CRC cells.

### CHK1 phosphorylation positively correlates with TRAF4 overexpression in CRC patient samples

**Fig. 6:**
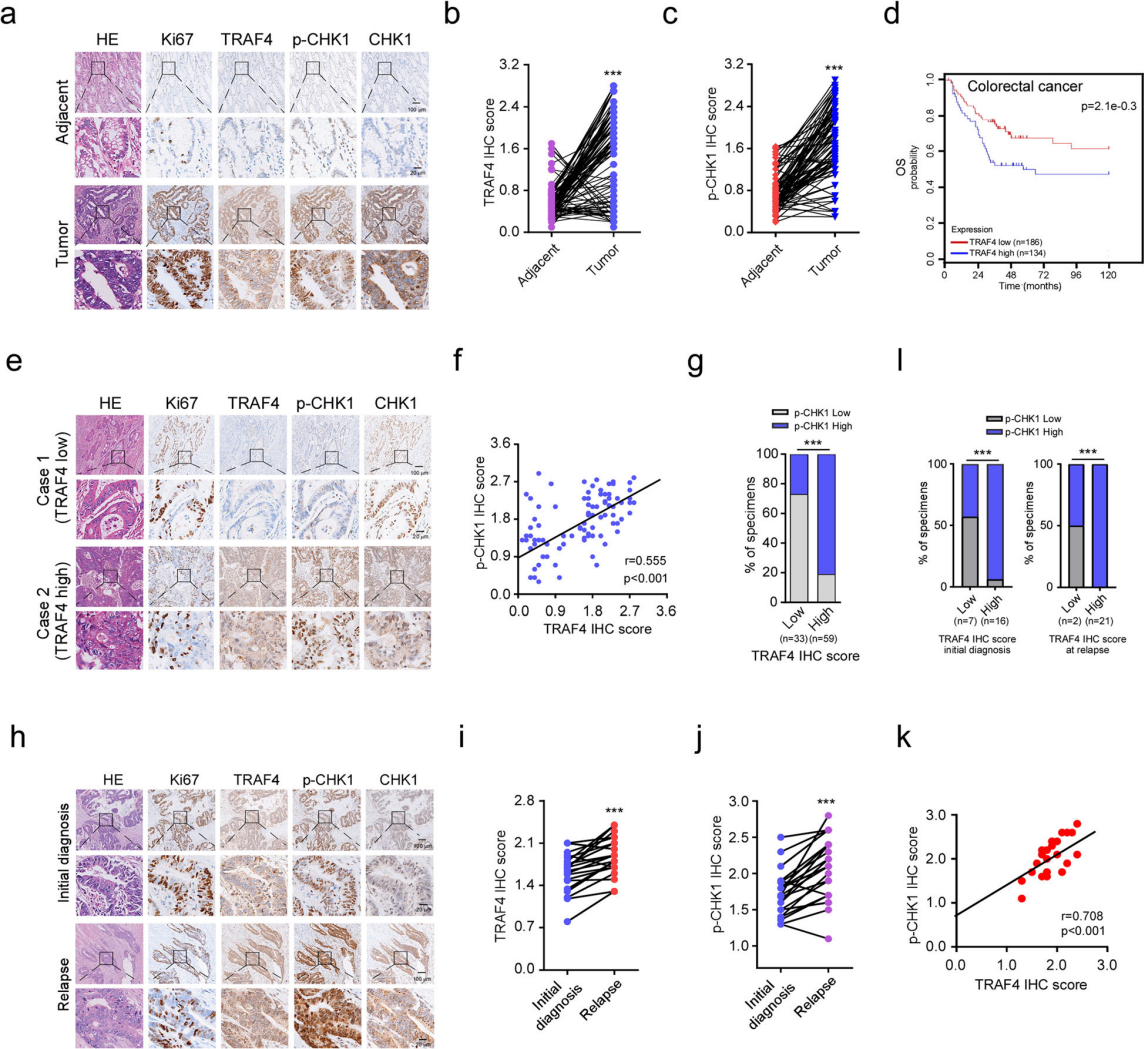
CHK1 phosphorylation positively correlates with TRAF4 overexpression in CRC patient samples. **a** Representative images of IHC staining for Ki67, TRAF4, p-CHK1 (S345), and CHK1 from 92 cases of primary tumors with matched adjacent tissues. **b, c** IHC scores for TRAF4 (**b**) and p-CHK1 (**c**) in 92 paired tumors and adjacent tissues. ***p < 0.001. **d** Kaplan–Meier curve shows the relationship between overall survival and TRAF4 expression in CRC patients. Data were obtained from the following online tool: https://hgserver1.amc.nl/cgi-bin/r2/main.cgi. **e** Representative images of IHC staining from a CRC primary tumor with high TRAF4 and p-CHK1 (S345) protein levels of or low TRAF4 and p-CHK1 (S345) protein levels. **f** The expression of TRAF4 and p-CHK1 (S345) is positively correlated. Pearson’s coefficient tests were performed to assess statistical significance. **g** The percentage of tissues displaying low or high TRAF4 and p-CHK1 (S345). ***p < 0.001. **h** Representative images of relapse tissues analyzed by IHC staining for Ki67, TRAF4, p-CHK1 (S345), and CHK1. **i, j** IHC scores of TRAF4 (**i**) and p-CHK1 (S345) (**j**) in paired primary and relapse samples. ***p < 0.001. **k** The expression of TRAF4 and p-CHK1 (S345) positively correlates in relapsed CRC tissues. Pearson’s coefficient tests were performed to assess statistical significance. **l** The percentage of specimens at initial diagnosis (left**)** and relapse (right) that display low or high TRAF4 and p-CHK1 (S345) levels after combination treatment. ***p < 0.001

To demonstrate the clinical relevance of our findings, we evaluated p-CHK1 (S345) and TRAF4 protein levels in 92 primary CRC tissues. The expression of TRAF4 was significantly increased in tumor tissues when compared to paired adjacent non-cancerous tissues (Fig. 6a, b). Notably, p-CHK1 (S345) was upregulated in colorectal tumor tissues (Fig. 6a, c). A greater percentage of tissues exhibited positive TRAF4 or p-CHK1 staining when tumor tissues were compared to adjacent non-cancerous tissues (Fig. S6a and S6b). Importantly, increased TRAF4 expression was associated with worse overall survival among CRC patients in our cohort or in one from the Human Protein Atlas (Fig. 6d, Fig. S6c). Representative images of low and high TRAF4 and p-CHK1 expression levels in tumor tissues from our cohort were shown in Fig. 6e. We found that TRAF4 protein levels were significantly and positively correlated with p-CHK1 levels (Fig. 6f). Among 92 patients, 59 had tumors with high TRAF4 staining, and 48 of these 59 cases also had high p-CHK1 staining (Fig. 6g). Both TRAF4 (Table S1) and p-CHK1 (Table S2) levels positively correlated with the clinical stage at the initial diagnosis (*p* < 0.01) and the lymph node status (*p* < 0.05).

The correlation between TRAF4-p-CHK1 signaling and sensitivity to chemotherapy was further confirmed in 23 cases of paired CRC specimens before chemotherapy and at relapse after 5-Fu + oxaliplatin + leucovorin combination therapy. The relapsed tumors exhibited significantly higher TRAF4 and p-CHK1 levels when compared to the paired untreated tumors. The expression of Ki67 was not different between the paired samples (Fig. 6h–j). Analysis of the pre- and post-treatment paired specimens from individual patients indicated that TRAF4 expression positively correlated with p-CHK1 expression in relapsed specimens (Fig. 6k, l). Collectively, these results suggest that the TRAF4-CHK1 signaling is associated with 5-Fu resistance in CRC.

### The CHK1 inhibitor overcomes chemoresistance in vitro and in vivo

**Fig. 7:**
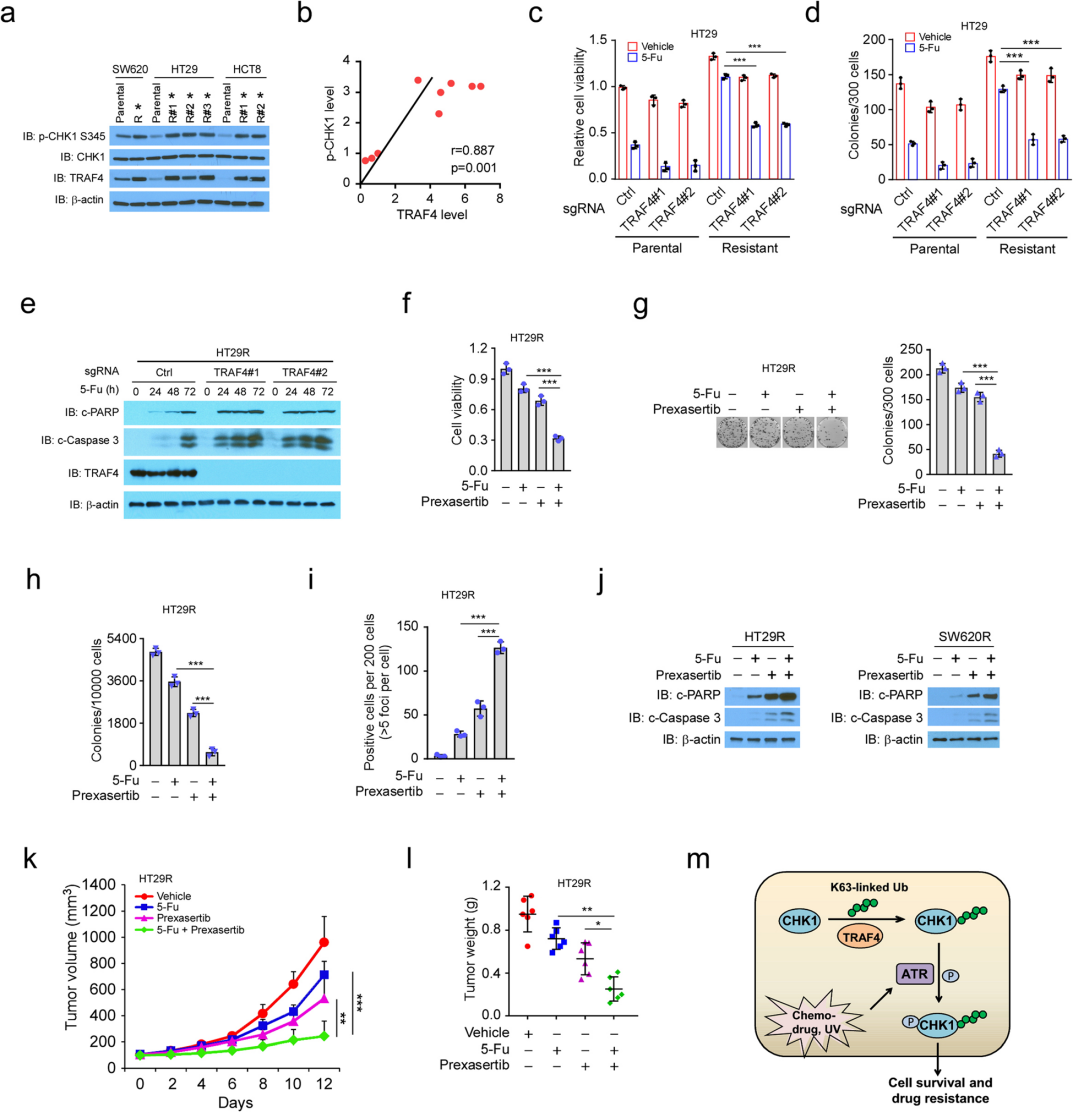
CHK1 inhibitor overcomes chemoresistance in vitro and in vivo. **a** IB analysis of TRAF4 and p-CHK1 in parental and chemoresistant CRC cells. The cell lysates of parental SW620, HT290, and HCT8 cells and 5-Fu-resistant CRC cells were treated with 5-Fu for 24 h, and WCEs were subjected to IB analysis. *5-Fu-resistant clones. **b** Correlation between TRAF4 and p-CHK1 in parental cells and 5-Fu-resistant clones displayed in **a**. Pearson’s coefficient tests were performed to assess statistical significance. **c** TRAF4 knockout enhances the sensitivity of HT29 cells to 5-Fu. HT29 parental cells and 5-Fu-resistant (HT29R) cells were transfected with control or TRAF4 sgRNA and treated with 100 μM 5-Fu for 72 h, followed by MTS analysis. ***p < 0.001. **d**, TRAF4 knockout reduces colony formation in HT29 cells treated with 5-Fu. The stable cells generated in **c** were treated with 100 μM 5-Fu for 24 h and subjected to a plate colony formation assay. ***p < 0.001. **e**, IB analysis of apoptosis-related proteins in HT29R stable cells treated with 5-Fu. **f-i** Cell viability (**f**), plate colony formation assay (**g**), soft-agar assay (**h**), and IF detection of γ-H2AX (**i**) in HT29R cells treated with the vehicle control, 100 μM 5-Fu, the CHK1 inhibitor, prexasertib, or a 5-Fu + prexasertib combination. ***p < 0.001. **j** IB analysis of apoptosis-related proteins in HT29R and SW620R cells treated with the vehicle control, 5-Fu, prexasertib, or a 5-Fu + prexasertib combination. **k**, **l** Tumorigenesis of HT29R cells treated with 5-Fu and prexasertib. Tumor size was monitored (**k**), and tumors were weighed (**l**). *p < 0.05, **p < 0.01, ***p < 0.001. **m** A working model of TRAF4-mediated CHK1 ubiquitination and activation in cancer cell chemotherapy resistance

To determine the relationship between TRAF4–p-CHK1 signaling and chemotherapy resistance in CRC, we constructed 5-Fu-resistant cells using three human CRC cell lines: SW620, HT29, and HCT8. Daughter cell clones with IC_50_ values at least 20-fold greater than the parental cells were deemed resistant. TRAF4 and p-CHK1 were significantly upregulated in cells resistant to 100 μM 5-Fu, when compared to the parental cell lines (Fig. 7a). Moreover, TRAF4 expression positively correlated with p-CHK1 expression in the 5-Fu-resistant CRC cells (Fig. 7b). To determine whether a high level of TRAF4 was required for maintaining chemotherapy resistance, we generated stable TRAF4-knockout cells in two 5-Fu-resistant cell lines: HT29R and SW620R. High concentrations of 5-Fu substantially attenuated cell viability (Fig. 7c, Fig. S7a) and colony formation (Fig. 7d, Fig. S7b) in the parental HT29 and SW620 cells, both with or without TRAF4. However, the inhibitory effects were only observed in the TRAF4-knockout HT29R and SW620R cells. No inhibition was present in the TRAF4-WT HT29R or the SW620R cells. Treatment with 5-Fu robustly increased the cleavage of PARP and caspase 3 in TRAF4 knockout-resistant cells, but the effect was less pronounced in TRAF4 WT-resistant cells (Fig. 7e, Fig. S7c). These results indicate the apoptosis pathway was activated after 5-Fu treatment.

To determine whether overexpression of TRAF4-mediated chemoresistance was associated with CHK1 activation in CRC cells, we examined the efficacy of a CHK1 inhibitor, prexasertib, in abrogating 5-Fu resistance. Although 5-Fu or prexasertib alone partially decreased cell viability in HT29R (Fig. 7f**)** and SW620R (Fig. S7d) cells, the combination therapy enhanced the anti-tumor effects. Similar results were also observed for anchorage-dependent and -independent colony formation (Fig. 7g, h; Fig. S7e and S7f). Consistently, prexasertib treatment promoted 5-Fu-induced γ-H2AX expression (Fig. 7i) and increased the cleavage of PARP and caspase 3 in HT29R cells (Fig. 7j). Xenograft tumors that were derived from TRAF4-knockout HT29R cells and treated with 5-Fu exhibited a significant decrease in growth and weight when compared to tumors derived from HT29R cells with WT-TRAF4 (Fig. S7g and S7h). The combination of 5-Fu and prexasertib synergistically reduced tumor growth and weight when compared to each of the single agent treatments (Fig. 7k, l). Together, these results suggest that the TRAF4-p-CHK1 signaling plays a critical role in 5-Fu resistance and that a combination of 5-Fu with CHK1 inhibitors could be a promising strategy to overcome chemoresistance in CRC cells.

## Discussion

DDR signaling is orchestrated by the ATM–CHK2 pathway, which is activated by double-strand DNA breaks (DSBs), and the ATR-CHK1 pathway, which is activated by tracts of single-strand DNA (ssDNA). DSBs activate not only ATM, but also ATR as the processing of DSBs generates replication protein A-coated ssDNA and recruits ATR. ATR then subsequently phosphorylates and activates CHK1 [25]. CHK1 activation is largely regulated through post-translational modification, including phosphorylation and ubiquitination [26, 27]. Phosphorylation not only activates CHK1, but also promotes its K48-linked ubiquitination and degradation after prolonged replication stress [14, 15]. The E3 ligases cullin 1, cullin 4, and F box protein 6 ubiquitinate and degrade CHK1 to terminate its activity, whereas deubiquitination of CHK1 by USP1, USP7, and ATX3 reverses this termination [14, 28–30]. In contrast to K48-linked ubiquitination that promotes target degradation, K63-linked ubiquitination serves as a molecular platform for protein–protein interaction, which is required for kinase activation, protein trafficking, receptor endocytosis, and DNA damage repair [31]. Accumulating evidence suggests a critical role of K63-linked polyubiquitination in the regulation of kinase activation. The E3 ligase TRAF6-, TRAF4-, and Skp2-mediated K63-linked ubiquitination of AKT is required for AKT activation in human cancer cells [16, 32–34]. Skp2-dependent ubiquitination and activation of LKB1 in hepatocellular carcinoma cells is essential for cancer cell survival under energy stress [35]. Furthermore, Skp2 catalyzes K63-linked ubiquitination of NBS1, promoting for the interaction between NBS1 and ATM and thereby facilitating ATM recruitment to the DNA foci for activation [36]. In this study, we show that in response to the DDR, CHK1 ubiquitination on K132 is catalyzed by TRAF4, which is required for CHK1 chromatin association, ATR binding, and subsequent phosphorylation and activation. CHK1 phosphorylation and activation by ATR are blocked in TRAF4-deficient CRC cell lines. Importantly, TRAF4 deficiency significantly decreases the proliferation, colony formation, and tumorigenesis of cancer cells under treatment with chemotherapeutic agents.

Previous reports showed that CHK1 chromatin association and activation are regulated dynamically and cell- and context-specific. For example, in 293T cells, SPRTN proteolysis releases CHK1 from the sites of replicative chromatin at the S stage during DNA replication [37]. However, in U2OS cells, DNA damage-induced a PIKK-dependent release of CHK1 from chromatin [38]. Phosphorylation of S317, but not S345, is required for CHK1 disassociation from chromatin in DDR [39]. In this study, we show that TRAF4-mediated CHK1 K132 ubiquitination is critical in CHK1 chromatin binding and ATR-induced phosphorylation. Furthermore, we show that CHK1 ubiquitination by TRAF4 is independent of ATR-mediated CHK1 S317/345 phosphorylation, indicating that the K63-linked ubiquitination is required for CHK1 activation at a very early stage of DDR. This is in line with a previous report demonstrating that removing the K63-linked ubiquitination chains by the deubiquitinase USP3 facilitates the disassociation of CHK1 from chromatin after CHK1 phosphorylation by ATR [40]. These results suggest a novel intermediate step for the well-established ATR-CHK1 signaling during the DDR: TRAF4 ubiquitinates CHK1 on K132, which is a prerequisite for CHK1 phosphorylation and activation by ATR (Fig. 7m).

TRAF4 is overexpressed in many cancer types [41, 42]. As an E3 ligase, TRAF4 can either degrade or activate targets through different ubiquitination linkages [16–18]. TRAF4 catalyzes K48-linked ubiquitination of SMURF2 to downregulate SMURF2 and promote breast cancer metastasis [17]. TRAF4 mediates K63-linked ubiquitination of AKT to stimulate AKT membrane recruitment and activation [16]. In addition, K27- and K29-linked ubiquitination of TrkA, a neurotrophin receptor tyrosine kinase, by TRAF4 increases TrkA kinase activity to enhance prostate cancer metastasis [18]. The present study identifies CHK1 as a novel substrate of TRAF4 that participates in the DDR and pro-survival signaling in multiple cancer cells by orchestrating K63-linked ubiquitination of CHK1. This work significantly expands our understanding of the substrates and functions of TRAF4 in DDR and cancer cell survival. An adaptor protein BTG3 and E3 ligase CRL4^Cdt2^ were recently identified to regulate CHK1 ubiquitination [13] in different ways from TRAF4. First, TRAF4 knockdown causes a larger reduction in CHK1 ubiquitination than BTG3 knockdown [13]. Second, BTG3 acts as a tumor suppressor that is required for CHK1 ubiquitination and maintaining genomic stability. In contrast, high expression of TRAF4 enhances CHK1 activation and confers cancer cell chemoresistance, which suggests that TRAF4 functions as an oncogene. Finally, K132 in CHK1 is identified as a conserved residue that is ubiquitinated by TRAF4 in multiple cell lines; however, there is minimal evidence that CRL4^Cdt2^ catalyzes CHK1 ubiquitination at this site.

Most chemotherapeutics induce the DDR and apoptotic cell death. 5-Fu inhibits thymidylate synthetase, reducing the size of the available dNTP pools and therefore increasing the stalling of replication forks. Topoisomerases control DNA supercoiling and entanglement by catalyzing nicking and re-ligation of DNA strands. Topoisomerase inhibitors like irinotecan form a complex with topoisomerases and DNA, physically hindering ongoing replication forks and eventually causing cell death [43]. It is widely believed that cancer cells maintain a proficient checkpoint system, including the ATR-CHK1 pathway, which, upon DNA damage-based therapies, halts cell cycle and allows DNA repair, thus promotes cell survival and confers therapeutic resistance [44]. The balance between DNA repair and cell apoptosis determines whether cancer cells live or die after chemotherapy. Checkpoint kinases play pivotal roles in maintaining this balance. Suppression of CHK1 in cancer cells impairs the DDR and DNA repair, which eventually potentiates the cell-killing effect of chemotherapeutic drugs [26], enhances the anti-tumor effect of PD-L1 blockade, and augments cytotoxic T-cell infiltration in vivo [45]. On the other hand, hyperactivation of ATR-CHK1 signaling confers chemotherapeutic resistance in multiple tumor models [7, 46, 47]. CHK1 inhibitors, including prexasertib and MK-8776 [48, 49], are currently in clinical trials alone or in combination with cytotoxic agents, such as cisplatin, pemetrexed, 5-Fu, and gemcitabine, to treat both solid tumors and hematological malignancies [11, 50–55]. In this study, we show that CHK1 phosphorylation positively correlates with TRAF4 in 5-Fu-resistant CRC cells and in relapsed CRC samples. TRAF4 drives chemoresistance, primarily through CHK1 activation in a ubiquitination-dependent manner in CRC cells. In TRAF4-deficient CRC cells, CHK1 is not phosphorylated or activated by 5-Fu treatment, which results in impaired chromosome alignment, increased premature mitosis, and polyploidy. In contrast, in TRAF4 proficient CRC cells, blocking CHK1 ubiquitination with a K132R mutation sensitizes CRC cells to chemotherapy in vitro and in vivo. In addition, the combination of a CHK1 inhibitor and 5-Fu enhances chemotherapeutic-induced cell death and attenuates xenograft tumor development.

## Conclusions

In summary, K63-linked ubiquitination of CHK1 by TRAF4 is a prerequisite step for CHK1 phosphorylation and activation by ATR upon DNA damage and likely drives chemoresistance in CRC cells. TRAF4 overexpression and CHK1 phosphorylation are associated with poor prognosis, treatment resistance, and disease recurrence in CRC patients. Thus, targeting the TRAF4-CHK1 signaling is a promising strategy for treating chemoresistant CRC and beyond.

## Supplementary information

**Additional file 1.** Supplementary Figures

**Additional file 2.** Supplementary Tables

## Data Availability

All data and materials supporting the conclusion of this study have been included within the article and the supplemental data.

## Abbreviations

CRC: Colorectal cancer
5-Fu: Fluorouracil
DDR: DNA damage response
ATM: Ataxia telangiectasia mutated
ATR: ATM- and Rad3-related
CHK1: Checkpoint kinase 1
RING: Really interesting new gene
TRAF4: Tumor necrosis factor receptor-associated factor 4
DMSO: Dimethyl sulfoxide
IB: Immunoblotting
IP: Immunoprecipitation
RIPA: Radioimmunoprecipitation assay
BCA: Bicinchoninic acid
PBS: Phosphate-buffered saline
CRISPR: Clustered regularly interspaced short palindromic repeats
SDS-PAGE: Sodium dodecyl sulfate–polyacrylamide gel electrophoresis
IHC: Immunohistochemical
H&E: Hematoxylin and eosin
UV: Ultraviolet
WT: Wild type
IF: Immunofluorescence
EdU: 5-ethynyl-2′-deoxyuridine
IC_50_: Half maximal inhibitory concentration
DSB: Double-strand DNA break
