# Supplementary material for: Ubiquitination of the DNA-damage checkpoint kinase CHK1 by TRAF4 is required for CHK1 activation
Source: J Hematol Oncol. 2020 May 1;13:40. doi: 10.1186/s13045-020-00869-3 (PMC7193419; doi:10.1186/s13045-020-00869-3)
Supplement: Supplementary file 1 — Additional file 1. Supplementary Figures [file 13045_2020_869_MOESM1_ESM.pdf]

Figure S1

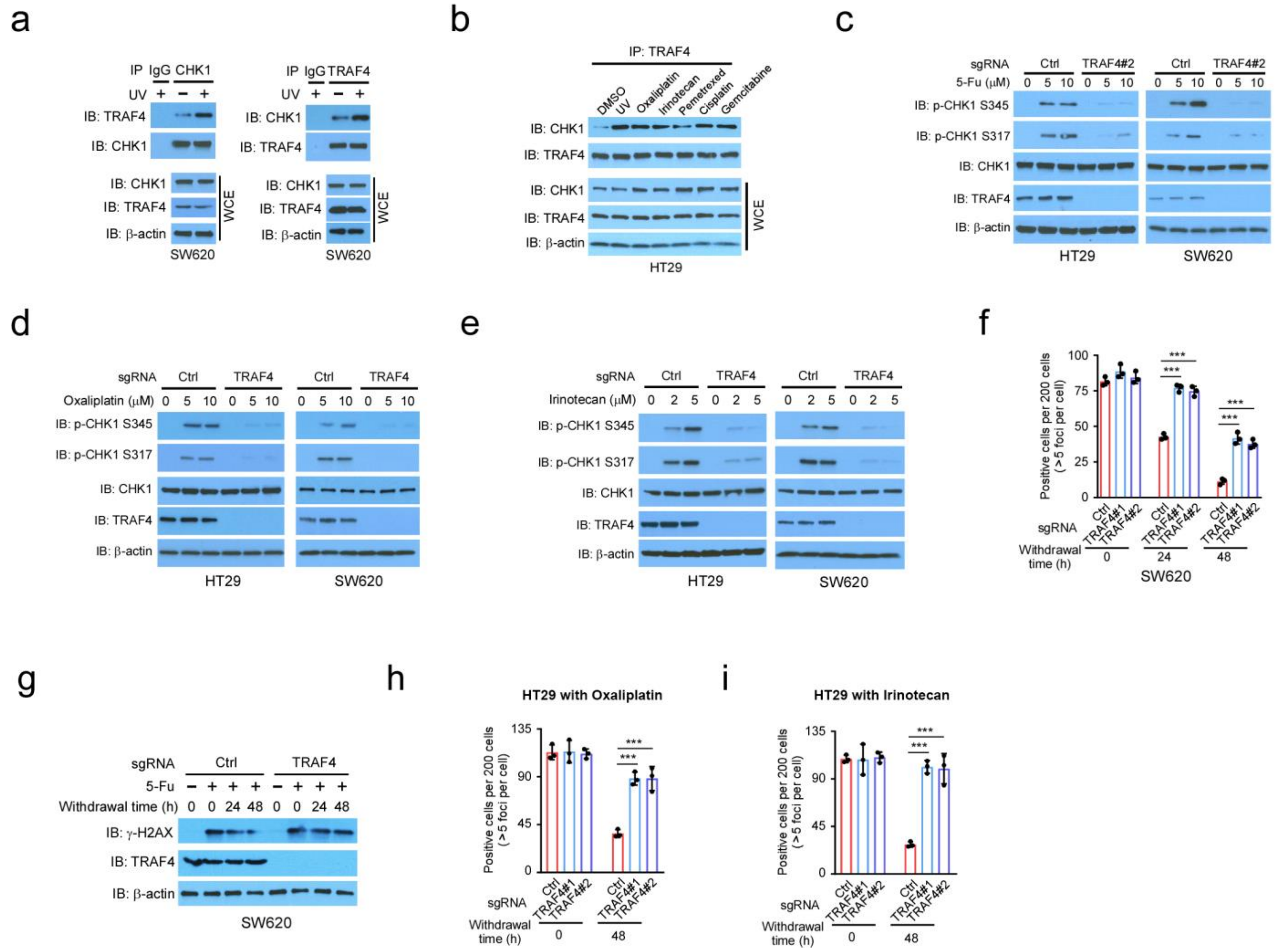

Supplementary Figure 1. TRAF4 promotes CHK1 activation in response to DNA damage. a, Co-IP analysis of the endogenous TRAF4-CHK1 interaction in SW620 cells. Cells were treated with UV, the whole-cell extracts (WCEs) were collected and subjected to a Co-IP assay and immunoblotting (IB) analysis. **b**, Co-IP analysis of endogenous TRAF4 and CHK1 interactions in HT29 cells. Cells were treated with chemotherapeutic agents, the WCEs were collected and subjected to Co-IP and IB analysis. UV treatment served as a positive control. **c**, Knockout of TRAF4 reduces CHK1 phosphorylation in 5-Fu-treated CRC cells. HT29 and SW620 cells expression sgCtrl or sgTRAF4 were treated with 5-Fu for 24 h, WCE was subjected to IB analysis. **d and e**, Knockout of TRAF4 reduces CHK1 phosphorylation in oxaliplatin- (**d**), or irinotecan-treated (**e**) HT29 and SW620 cells. **f** and **g**, TRAF4 knockout compromises DNA repair in 5-Fu-treated SW620 cells. TRAF4-WT or TRAF4-knockout SW620 cells were treated with 5-Fu for 24 h, then maintained in 5-Fu-free medium and subjected to IF (**f**) or IB (**g**) analysis at various time points with γ-H2AX antibody. ***p < 0.001. **h** and **i**, TRAF4 knockout compromises DNA repair in oxaliplatin- (**h**) or irinotecan- (**i**) treated HT29 cells. TRAF4-WT or TRAF4-knockout HT29 cells were treated with oxaliplatin (**h**) or irinotecan (**i**) for 24 h, then maintained

in fresh medium without chemotherapeutic agents and subjected to IF analysis at various time points with the γ-H2AX antibody. ***p < 0.001.

Figure S2

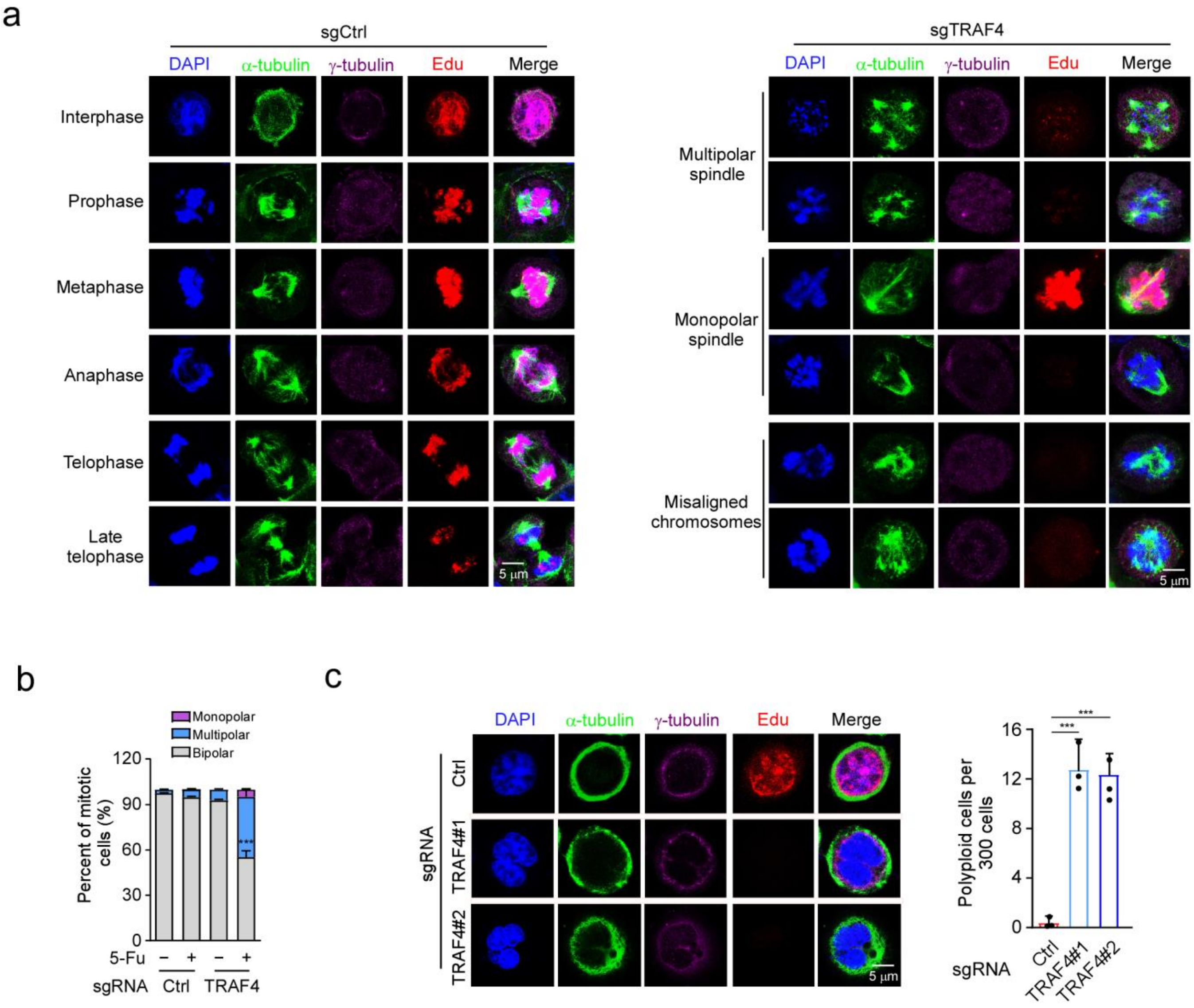

Supplementary Figure 2. TRAF4 knockout results in 5-Fu-induced defects in mitosis and the formation of polyploidy HT29 cells. a and **b**, TRAF4 knockout results in defects in mitosis. TRAF4-WT or TRAF4-knockout HT29 cells were treated with 5-Fu for 24 h. Cells were maintained in fresh medium containing 10 μM 5-ethynyl-2'-deoxyuridine (EdU) for an additional 24 h and fixed for IF analysis with α-tubulin and γ-tubulin antibodies (**a**). Quantification analysis of mitotic cells from **a** with a bipolar, multipolar, or monopolar spindle (**b**). ***p < 0.001. **c**, TRAF4 knockout leads to the formation of polyploidy cells. TRAF4-WT or TRAF4-knockout HT29 cells were treated with 5-Fu for

24 h. Cells were maintained in fresh medium containing 10 μM EdU for an additional 24 h and fixed for IF analysis with α-tubulin and γ-tubulin antibodies, 5-Fu-induced polyploidy cells were quantified by cell counting. A total of 300 cells for each experiment were counted, and the result was shown from three independent experiments. ***p < 0.001.

Figure S3

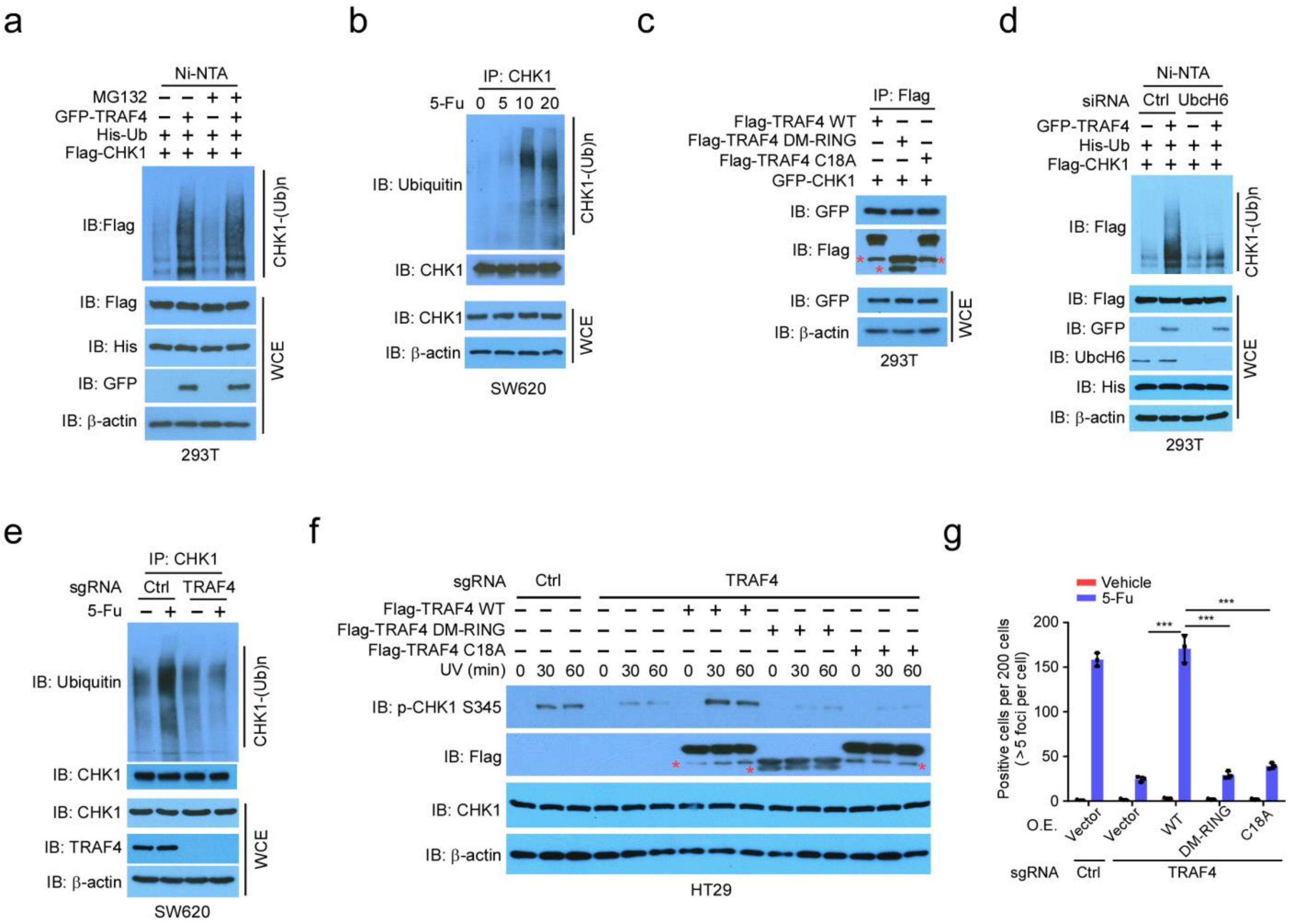

Supplementary Figure 3. TRAF4 is required for CHK1 K63-linked ubiquitination. **a**, *In vivo* ubiquitination assay in 293T cells transfected with various constructs and treated with MG132. **b**, 5-Fu promotes endogenous CHK1 ubiquitination in SW620 cells. **c**, 293T cells were transfected with the constructs and subjected to Co-IP analysis of CHK1 and TRAF4 mutant interactions. *non-specific signal. **d**, Knockdown of UbcH6 inhibits TRAF4-mediated CHK1 ubiquitination. 293T cells were transfected with various constructs and siRNA, and the cell lysates were subjected to an *in vivo* ubiquitination assay. **e**, Knockout of TRAF4 impairs 5-Fu-induced endogenous CHK1 ubiquitination in SW620 cells. **f**, CHK1 phosphorylation in TRAF4-knockout cells is rescued by exogenous WT TRAF4. TRAF4-knockout HT29 cells were transfected with various constructs and treated with UV, WCEs were collected at different time points for IB analysis. **g**, IF analysis of CHK1 phosphorylation in 5-Fu-treated

HT29 cells. TRAF4-WT or TRAF4-knockout HT29 cells were transfected with various constructs and treated with 5-Fu for 24 h. Cells were fixed and subjected to IF analysis with p-CHK1 (S345) antibody. ***$p < 0.001$.

Figure S4

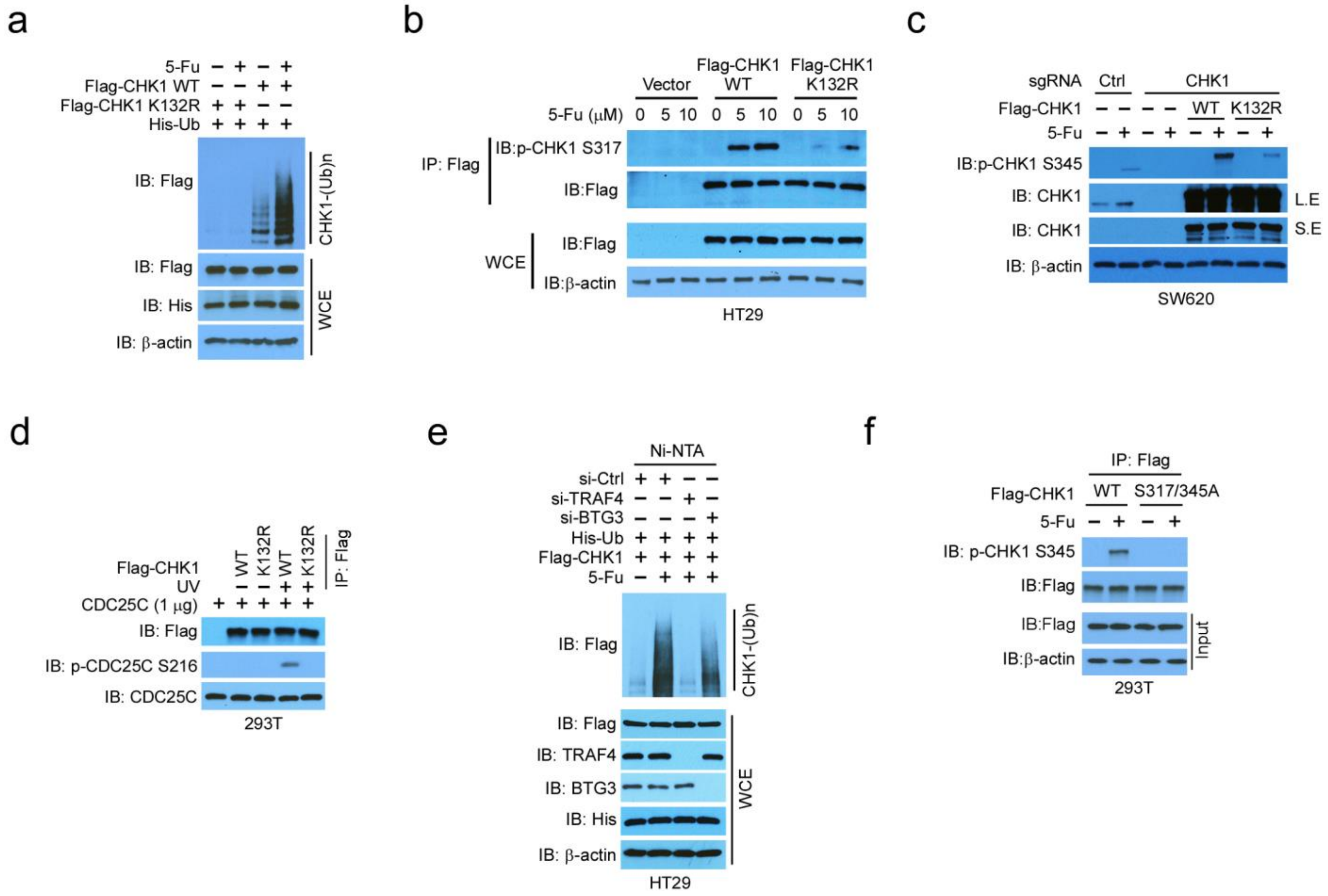

Supplementary Figure 4. TRAF4 promoted K132 ubiquitination is required for CHK1 activation. **a**, *In vivo* ubiquitination assay in HT29 cells transfected with various constructs and treated with 5-Fu. **b**, The K132R mutation impairs CHK1 phosphorylation in HT29 cells. HT29 cells were transfected with different plasmids and treated with 5-Fu. WCEs were immunoprecipitated and subjected to IB analysis. **c**, CHK1 phosphorylation in CHK1-null SW620 cells is rescued by exogenous WT CHK1. CHK1 WT or CHK1 knockout SW620 cells transfected with CHK1 WT or CHK1 K132R mutant were treated with 5-Fu and subjected to IB analysis. **d**, *In vitro* kinase assay of the CHK1 WT and K132R mutant. 293T cells were transfected with various constructs and treated with 5-Fu. WCEs were extracted and immunoprecipitated with Flag-tag antibody. The IP proteins were incubated with Cdc25C and subjected to an *in vitro* kinase assay. **e**, Knockdown of TRAF4 or BTG3 inhibits CHK1 ubiquitination in HT29

cells. HT29 cells transfected with siRNA and various constructs followed by 5-Fu treatment, WCE was subjected to *in vivo* ubiquitination assay. **f**, Phosphorylation of CHK1 WT and the S317/345 mutant upon 5-Fu treatment in 293T cells.

Figure S5

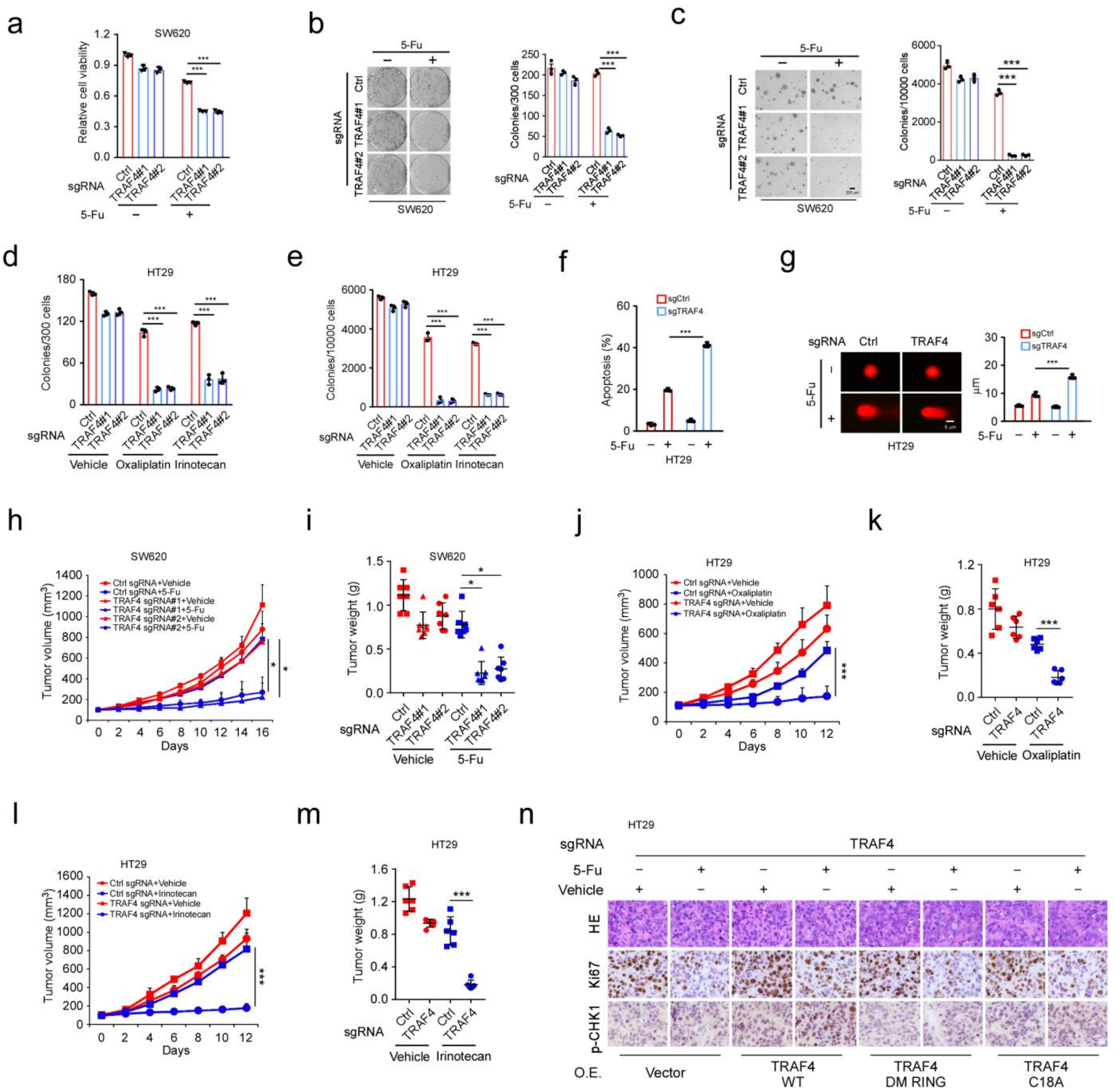

Supplementary Figure 5. Depletion of TRAF4 heightens the anti-tumor effect of chemotherapeutic agents in CRC. a, TRAF4 knockout reduces cell viability in SW620 cells with 5-Fu treatment. SW620

cells expression sgCtrl or sgTRAF4 were treated with 5-Fu for 72 h and analyzed with MTS assay. ***p < 0.001. **b** and **c**, Anchorage-dependent (**b**) and –independent (**c**) growth of TRAF4-WT or TRAF4-knockout SW620 cells with 5-Fu treatment. ***p < 0.001. **d** and **e**, Anchorage-dependent (**d**) and –independent (**e**) growth of TRAF4-WT or TRAF4-knockout HT29 cells with oxaliplatin and irinotecan treatment. ***p < 0.001. **f,** TRAF4 knockout enhances 5Fu-induced apoptosis. Flow cytometry analysis of apoptotic cells in 5-Fu-treated HT29 stable cells. **g,** TRAF4 knockout enhances 5Fu-induced DNA damage. Comet assay analysis of DNA damage in 5-Fu-treated HT29 stable cells. **h** and **i**, TRAF4 knockout enhances the efficacy of 5-Fu *in vivo*. TRAF4-WT or TRAF4-knockout SW620 cells were injected into NSG mice to create xenografts and mice then were treated with vehicle control or 5-Fu. Tumor sizes were monitored (**h**), and the tumor masses were weighed (**i**). *p < 0.05. **j** and **k**, TRAF4 knockout enhances the efficacy of oxaliplatin in HT29 xenografts *in vivo*. Tumor size was monitored (**j**), and the tumors were weighed at the study endpoint (**k**). ***p < 0.001. **l** and **m**, TRAF4 knockout enhances the efficacy of irinotecan *in vivo.* TRAF4-WT or TRAF4-knockout HT29 cells injected into NSG mice to create xenografts and mice then were treated with vehicle control or irinotecan. Tumor size was monitored (**l**), and the tumors were weighed (**m**). ***p < 0.001. **n**, TRAF4 reintroduction into TRAF4-null HT29 cells rescues tumorigenesis under 5-Fu treatment. TRAF4 WT or E3 ligase activity deficient mutants were reintroduced into TRAF4-null HT29 cells and injected into NSG mice to establish the xenograft mouse model. Ki67 and p-CHK1 were examined via IHC staining.

Figure S6

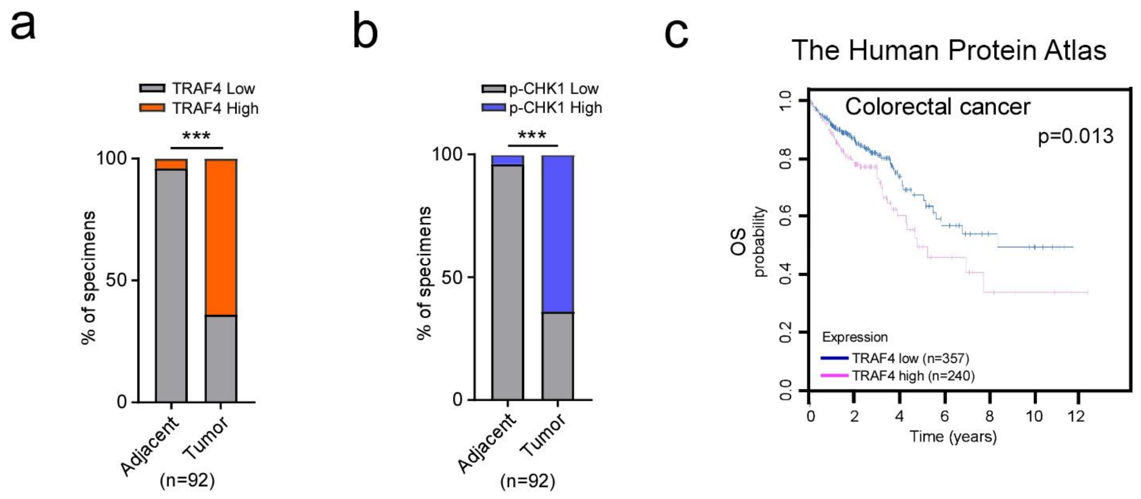

Supplementary Figure 6. TRAF4 overexpression correlates with CHK1 phosphorylation and poor prognosis in CRC patients. a, Percentage of adjacent and tumor tissues from 92 patients with CRC

displaying low or high TRAF4 expression. ***p < 0.001. **b**, Percentage of adjacent and tumor tissues displaying low or high p-CHK1 (S345) levels. ***p < 0.001. **c**, Kaplan–Meier curves show the relationship between overall survival and TRAF4 expression in CRC patients. Data were obtained from the Human Protein Atlas (https://www.proteinatlas.org).

Figure S7

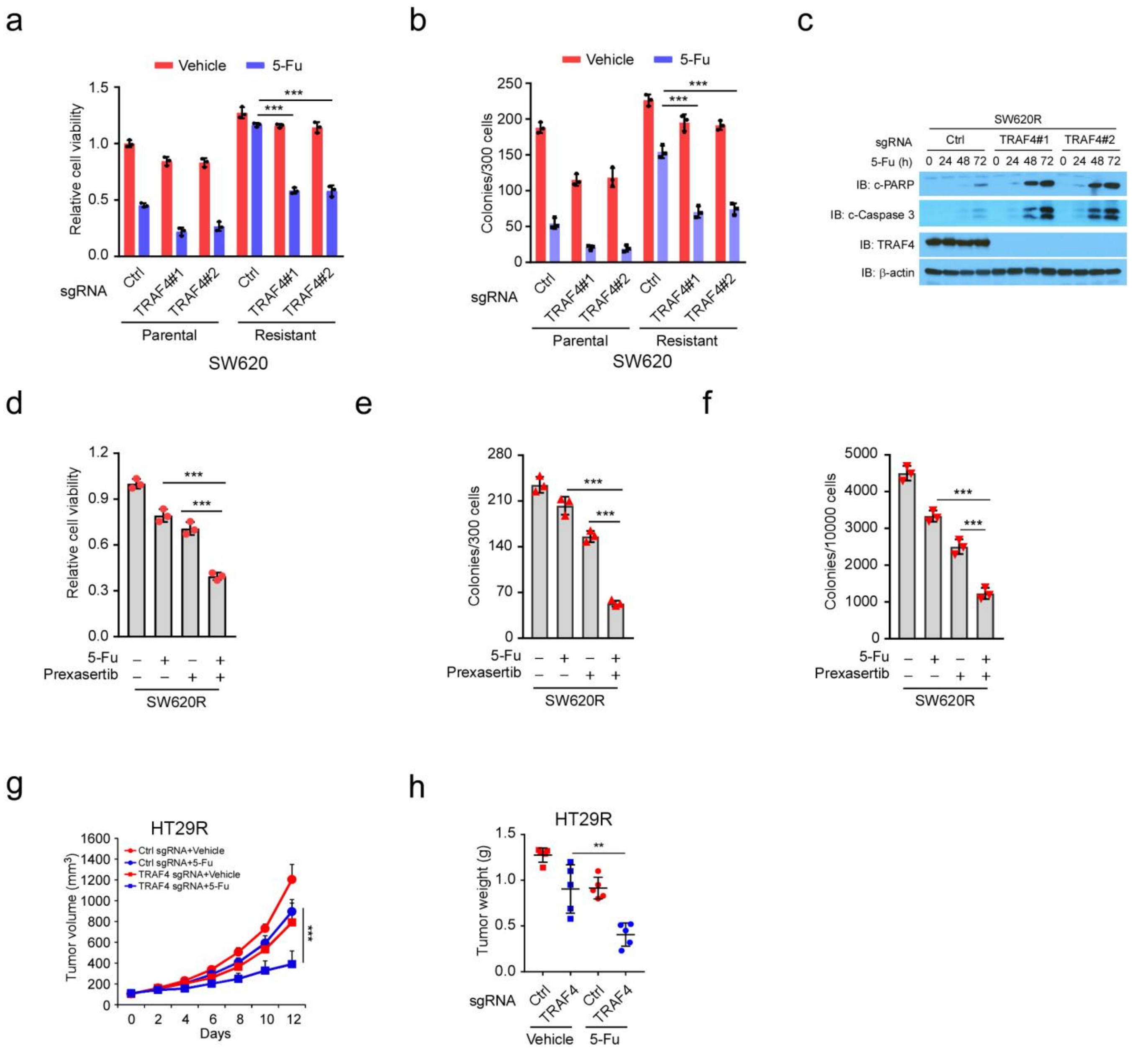

Supplementary Figure 7. CHK1 inhibitor sensitizes CRC cells to chemotherapy. a, TRAF4 knockout enhances the sensitivity of SW620 cells to 5-Fu. SW620 parental cells and 5-Fu-resistant (SW620R) cells were transfected with control or TRAF4 sgRNA and selected with puromycin to

construct stable cell lines. The generated control and TRAF4-knockout stable cells were treated with 100μM 5-Fu for 72 h and subjected to an MTS assay. ***$p < 0.001$. **b**, TRAF4 knockout reduces colony formation in SW620 cells treated with 5-Fu. The stable cells generated in **a** were treated with 100μM 5-Fu for 24 h and subjected to a plate colony-formation assay. ***$p < 0.001$. **c**, IB analysis of apoptosis-related proteins in SW620R stable cells treated with 100μM 5-Fu. **d–f**, Cell viability (**d**), anchorage-dependent (**e**) and -independent (**f**) cell growth of SW620R cells treated with vehicle control, 100μM 5-Fu, prexasertib, and the combination 5-Fu + prexasertib. ***$p < 0.001$. **g** and **h**, Tumorigenesis of HT29R cells expression sgCtrl or sgTRAF4 treated with 5-Fu. TRAF4-WT or TRAF4-knockout HT29R cells injected into NSG mice to create xenografts and mice then were treated with vehicle control or 5-Fu. Tumor size was monitored (**g**), and tumors were weighed (**h**). **$p < 0.01$, ***$p < 0.001$.
