# Supplementary material for: Ubiquitination of the DNA-damage checkpoint kinase CHK1 by TRAF4 is required for CHK1 activation
Source: J Hematol Oncol. 2020 May 1;13:40. doi: 10.1186/s13045-020-00869-3 (PMC7193419; doi:10.1186/s13045-020-00869-3)
Supplement: Supplementary file 2 — Additional file 2. Supplementary Tables [file 13045_2020_869_MOESM2_ESM.pdf]

Table 1. TRAF4 protein expression in colon cancer tissue and adjacent tissue.

**Tissue**	**Number of Cases**	**TRAF4**		**p-value**
		**Low**	**High**	
Adjacent	92	88	4	0.000
Tumor	92	33	59	

Chi-square test.

$p<0.05$ indicates a significant association among the variables.

Table 2. p-Chk1 protein expression in colon cancer tissue and adjacent normal tissue.

**Tissue sample**	**Number of Cases**	**p-Chk1**		**p-value**
		**Low**	**High**	
Adjacent	92	87	5	0.000
Tumor	92	40	52	

Chi-square test.

$p<0.05$ indicates a significant association among the variables.

Table 3. Relationships between the expression of TRAF4 and clinical pathological characteristics in colon cancer patients.

**Characteristics**	**Number of Cases**	**Expression of TRAF4**		**p-value**
		**Low (n=33)**	**High (n=59)**	
**Gender**				0.455
Male	51	20	31	
Female	41	13	28	
**Age (years)**				0.151
≤60	30	9	21	
>60	62	24	38	
**Initial clinical stage**				0.000
≤IIa	48	28	20	
>IIa	44	5	39	
**Tumor stages**				0.780
T1+T2	29	11	18	
T3+T4	63	22	41	
**Lymph node status**				0.000
N0 (negative)	50	28	22	
N1 or above (positive)	42	5	37	

Chi-square test.

$p<0.05$ indicates a significant association among the variables.

Table 4. Relationships between the expression of p-CHK1 and clinical pathological characteristics in colon cancer patients.

		Expression of p-CHK1		p-value
**Characteristics**	**All cases**	**Low (n=35)**	**High (n=57)**	
**Gender**				0.267
Male	51	20	31	
Female	41	15	26	
**Age (years)**				0.850
≤60	30	11	19	
>60	62	24	38	
**Initial clinical stage**				0.004
≤IIa	48	25	23	
>IIa	44	10	34	
**Tumor stages**				0.988
T1+T2	29	11	18	
T3+T4	63	24	39	
**Lymph nodes status**				0.010
N0 (negative)	50	25	25	
N1 or above (positive)	42	10	32	

Chi-square test.

$p<0.05$ indicates a significant association among the variables.
